# CheRRI—Accurate classification of the biological relevance of putative RNA–RNA interaction sites

**DOI:** 10.1093/gigascience/giae022

**Published:** 2024-06-05

**Authors:** Teresa Müller, Stefan Mautner, Pavankumar Videm, Florian Eggenhofer, Martin Raden, Rolf Backofen

**Affiliations:** Bioinformatics Group, Department of Computer Science, University of Freiburg, Georges-Koehler-Allee 106, 79110 Freiburg, Germany; Bioinformatics Group, Department of Computer Science, University of Freiburg, Georges-Koehler-Allee 106, 79110 Freiburg, Germany; Bioinformatics Group, Department of Computer Science, University of Freiburg, Georges-Koehler-Allee 106, 79110 Freiburg, Germany; Bioinformatics Group, Department of Computer Science, University of Freiburg, Georges-Koehler-Allee 106, 79110 Freiburg, Germany; Bioinformatics Group, Department of Computer Science, University of Freiburg, Georges-Koehler-Allee 106, 79110 Freiburg, Germany; Bioinformatics Group, Department of Computer Science, University of Freiburg, Georges-Koehler-Allee 106, 79110 Freiburg, Germany; Signalling Research Centre CIBSS, University of Freiburg, Schaenzlestr. 18, 79104 Freiburg, Germany

**Keywords:** RNA–RNA interactome, direct duplex detection, classification, functional RRI, false positives

## Abstract

**Background:**

RNA–RNA interactions are key to a wide range of cellular functions. The detection of potential interactions helps to understand the underlying processes. However, potential interactions identified via *in silico* or experimental high-throughput methods can lack precision because of a high false-positive rate.

**Results:**

We present CheRRI, the first tool to evaluate the biological relevance of putative RNA–RNA interaction sites. CheRRI filters candidates via a machine learning–based model trained on experimental RNA–RNA interactome data. Its unique setup combines interactome data and an established thermodynamic prediction tool to integrate experimental data with state-of-the-art computational models. Applying these data to an automated machine learning approach provides the opportunity to not only filter data for potential false positives but also tailor the underlying interaction site model to specific needs.

**Conclusions:**

CheRRI is a stand-alone postprocessing tool to filter either predicted or experimentally identified potential RNA–RNA interactions on a genomic level to enhance the quality of interaction candidates. It is easy to install (via conda, pip packages), use (via Galaxy), and integrate into existing RNA–RNA interaction pipelines.

**Key Points**

- Classification of putative RNA–RNA interaction (RRI) sites concerning their biological relevance
- Filtering of false-positive RRIs
- Automated model building for custom interactome data

## Background

RNA–RNA interactions (RRIs) are fundamental for many cellular processes [1]. Noncoding RNAs (ncRNAs) regulate gene expressions on a transcriptional as well as posttranscriptional level, like long ncRNAs [2] or microRNAs (miRNAs) [3]. Bacterial small regulatory RNAs (sRNAs) regulate all kinds of cellular processes and are therefore studied on a genome-wide scale [4, 5]. Some ncRNAs are parts of complex regulation networks, which complicates their investigation [6].

The importance of RNA–RNA interactions has given rise to RNA–interactome databases like RISE [7] or RNAInter [8], providing immense datasets of interactions. These datasets, however, show a high diversity in terms of data source, quality, and reliability. Gong et al. [7] discussed that the overlap between RRIs is varying between different experimental protocols and also between different prediction methods.

A more unified picture of the RNA–interactome of a specific organism is provided by direct duplex detection (DDD) methods. They are based on high-throughput crosslinking structure analysis without using specific RNA-binding proteins and are a promising source of reliable transcriptome-wide experimental RRI data [9, 10]. Different protocols like PARIS [11], LIGR-seq [12], and SPLASH [13] are available, out of which PARIS was found to identify the highest number of RRI sites [14]. An RRI site, as identified by a DDD method, is defined by 2 crosslinked (short) subsequences but does not provide details about the occurring intermolecular base pairing. Since crosslinking is not always caused by true RRIs, DDD results are also listing false-positive RRI sites.

Another commonly used approach for detecting putative RNA–RNA interactions is the computational prediction of potential intermolecular base pairs. Typically, programs like RIblast [15], RIsearch2 [16], or IntaRNA [17] are used, while the latter was found to be among the most reliable of the available bioinformatics tools [18, 19] with flexibly adjustable constraints [20]. There are recent efforts to solve RRI predictions using machine learning methods [21–23]. These approaches are, however, typically tailored to a specific type of regulatory RNAs like miRNAs [24] or prokaryotic sRNAs [25]. To face the high amount of putative predictions and high false-positive rates, such methods often combine prediction with feature-based filtering (e.g., for the selection of potent siRNAs) [26]. While such filters are effective, they are currently tailored to and integrated into respective tools and cannot be applied on putative RRI sites derived from other sources.

So far, there exists no method-independent approach to filter already identified potential RRI prediction sites based on their biological relevance, where relevance should be based on distinguishing features learned from a curated dataset that is based on experimentally identified RNA–RNA interactome data. Such a filtering method would allow to postprocess experimental data to prune false positives resulting from wrong predictions or protocol artifacts.

**Figure 1:**
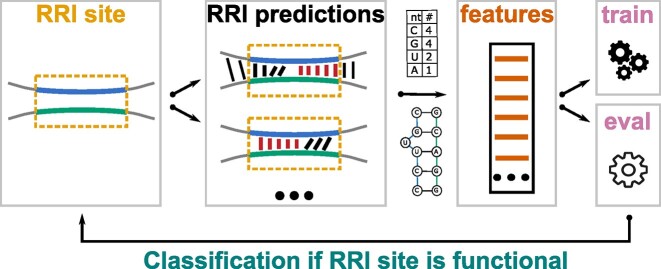
Graphical abstract. CheRRI takes RRI sites (yellow) as input and adds genomic context up- and downstream (gray). Inside these extended RRI sites, RRI predictions (black, red) are computed by IntaRNA. The RRI prediction can exceed the original RRI site (fine black), but the seed (red) needs to be within the RRI site. CheRRI then extracts various sequence, context, and graph features (orange). These features are used to train a predictive model (train mode) or to evaluate whether a given RRI site is biologically relevant (eval mode).

Here, we introduce CheRRI to fill this gap. To enable the creation of a reliable model, the CheRRI curation subpipeline applies several automated quality checks to extract from a given potential training dataset a subset of reliable RRI sites. Based on the reliable RRI sites, CheRRI derives features from interactome-constraint RRI predictions, as well as additional sequence, context, and graph features. For instance, also information about known binding sites of RNA-binding proteins (RBPs) can be incorporated into the model, since RBPs are known to influence the local structure of RNAs and thus their binding capacity [27]. The generated features are used to automatically learn the best classification model for a given training dataset and to provide a precomputed, high-quality RRI evaluation model. An overview of the approach is given in Fig. 1. Our CheRRI tool provides, after learning, an evaluation mode that allows to filter putative RRI sites. That way, we can remove false-positive RRI sites from data produced by both experimental or *in silico* methods.

## Approach

**Figure 2:**
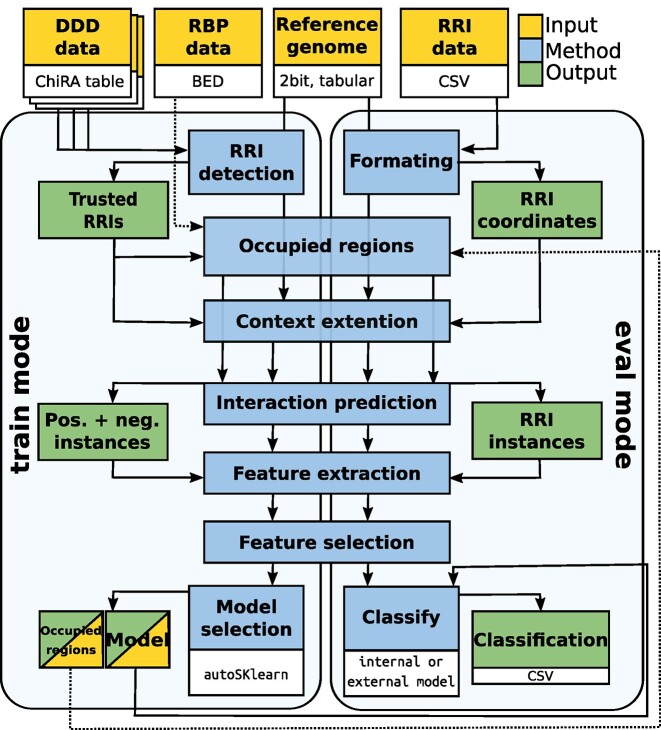
The CheRRI workflow. Except for the initial data-processing step, both the model selection step in train mode (left) and the classification step in eval mode (right) use the same core modules. In detail, the train mode takes DDD data as input while a tabular file containing RRI sites to be evaluated is provided in the eval mode. A reference genome can be automatically downloaded (human and mouse) or needs to be provided for both modes. Optionally, RBP data can be provided as well. After extracting the sequences with context, CheRRI uses IntaRNA to predict interactions anchored within the sites. Then various features are extracted from the predicted RRIs as well as sequence, context, and accessibility information. These features are then used to either build an organism-specific classification model (in train mode) or to evaluate (in eval mode) the given RRIs with such a model.

CheRRI uses genome-wide experimental RNA–RNA interactome data to train a classification model for a subsequent evaluation of putative RRI sites (see Fig. 1). CheRRI provides pretrained human (HEK293T cell line) and mouse (embryonic stem cells) models for its direct application to filter user-provided potential RRI sites in both organisms. To this end, the user only has to provide a set of putative RRI sites (i.e., genomic positions of both interacting subsequences). If not interested in the built-in human and mouse genomes, the corresponding reference genome is to be given as well. CheRRI is a pipeline built of different submodules and functions that are depicted in Fig. 2. A detailed functionality description is given in section S1 of the supplementary material and in the online documentation https://backofenlab.github.io/Cherri/.

To train a new evaluation model with CheRRI, a processed DDD dataset has to be provided. More precisely, it consists of sets of reliable RRI sites (i.e., pairs of short subsequences that were found to be interacting) in different replicates and, if possible, in concert with additional information like protein-binding sites. Thus, CheRRI is not restricted to a specific High Throughput Sequencing (HTS) interactome protocol or a dedicated chimeric read analysis pipeline. It solely needs the RRI site information. Details about data preparation, model training, and application are provided in the following sections.

### Preparing training via extraction of reliable RRI sites from interactome data

Our RRI site classification model is based on experimental RNA–RNA interactome data. In order to generate a reliable model, the provided data need to be pruned to “trustworthy” RRIs. This is done based on replicate and read analyses.

In case replicates are provided, only interactions present in all replicates are kept for further filtering and model generation. This is detected via an overlap comparison of the RRI sites. Boundary accuracy of detected RRI sites from different protocols can vary. Thus, based on preliminary analyses, we set the default overlap threshold to 30%. Technical details are provided in section S1.1 of the supplementary material.

To further enhance the dataset, we subsequently filter for reliable RRI sites via their Expectation Maximization (EM) scores, which can be computed with the chimeric read analyzer ChiRA [28]. We use a conservative default cutoff for the EM score (≥1), since we want to keep only sites that are highly reliable.

In summary, via the use of 2 filtering criteria—that is, (1) detection of RRI sites shared among replicates and (2) the selection of high-quality mapped reads—it is ensured that CheRRI’s training data are built from reliable RRI sites with strong evidence.

### Occupied regions within the genomic context of RRI sites

CheRRI also takes into account whether or not regions in the context of the RRI site of interest are likely occupied (i.e., involved in RNA–RNA or RNA–protein interaction). To this end, regions that are part of sites of the input interactome are considered occupied. The interactions used to build the occupied region library are selected on a lower score cutoff (≥0.5), to implement a very conservative definition of “occupied”.

Furthermore, the user can provide additional information of other putative binding sites. In detail, CheRRI supports the input of RBP binding site information in BED format. The respective regions are also added to the library of occupied regions that are excluded from interaction prediction in the next step.

### RRI base pairing details via constraint RRI prediction

Each RRI site will be represented by a set of features that are later assessed by the machine learning model. CheRRI takes both the sequence as well as hybridization characteristics of the RRI site and its surrounding genomic context into account. Thus, to investigate the hybridization strength of a putative interaction site, CheRRI explores multiple top-ranked IntaRNA RRI predictions per site. Investigating a set rather than a single prediction per site reduces the bias of the underlying thermodynamic model and incorporates structural alternatives and flexibility of the interaction. In detail, IntaRNA is constrained to identify the 5 most stable interactions that show a reliable subinteraction (of 5 consecutive base pairs, called a *seed*) covered by the 2 subsequences that define the RRI site. Thus, the predictions are anchored by the RRI site but can stretch over its limits into the context. Supplementary Table S1 gives an overview of the additional IntaRNA settings used by CheRRI, which can be adapted by the user.

**Figure 3:**
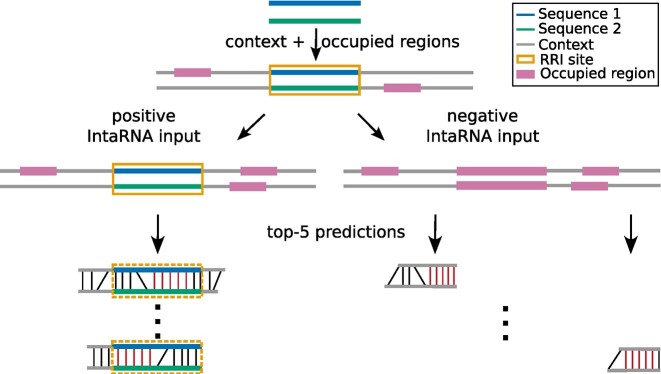
Generating interaction details for reliable RRI sites. Starting with the subsequences defining an RRI site (top blue and green) detected by a DDD method, the sequence is first extended with genomic context (gray). In these extended sequences, all regions known to be occupied (e.g., by interaction with other RNAs or proteins) are masked as occupied regions (pink boxes). To create interaction details for reliable interactions (positive data), IntaRNA predictions are required to show a seed (red base pairs) within the original RRI site (orange box) while avoiding masked regions. For negative interactions, also the RRI site is masked as occupied and “flanking” interactions from the RRI site’s genomic context are predicted. In both cases, the top 5 ranked IntaRNA predictions are subsequently taken into account to compile the features of an RRI site. The number of suboptimals can be changed by the user.

Furthermore, the interactions are also not allowed to cover known occupied regions, that is, regions that are either other RRI sites or known RBP binding sites (see above). Both constraints are depicted in the lower left of Fig. 3. This is important to give the computational tool the freedom to identify the most-stable base pairing patterns while linking the prediction with the experimental information with limited reliability concerning the site’s boundary information.

### Creating a sound negative dataset

In order to train and assess the power of our machine learning approach, we need besides the introduced reliable RRI data (our positive dataset) also a set of “noninteracting” data (negative data training). To ensure that our negative data show similar features as our positive dataset, negative training instances are predicted in close proximity to each positive instance but have to be outside of all other RRI sites. If available, additional interaction information like provided RNA–protein interaction sites is also masked to further enhance the generation of negative data with respective constraints. Negative interactions are then predicted within the not masked context areas as depicted in Fig. 3.

The resulting set of negative RRI predictions is neither overlapping with reliable RRI sites nor with known interaction sites but is likely showing sequence properties and thus interaction features similar to the positive set.

### Features, globally and on a neighborhood level

Various features of each IntaRNA prediction like interaction strength, accessibility, length, base pairing, and so on are subsequently taken into account. Further sequential features like sequence complexity, GC content, or other nucleotide ratios are generated. More complex sequence features like the sequence complexity of each interaction site as well as normalized features like the energy normalized by the interaction length or the number of GCs are also calculated to represent interactions. The list of features considered by CheRRI models is provided in Supplementary Table S2.

In addition to these global features, graph kernel features are available. Here a graph of all predicted base pairing patterns (multiple per site) is generated, which incorporates both sequence and structure information. From this, (sub)graph features are extracted via a Weisfeiler–Lehman message-passing scheme implemented in EDeN [29] (Explicit Decomposition with Neighborhoods), which allows for the essential link of local structural context (base pairing) with sequence information in RNAs.

### Automated selection of a good feature set and classification algorithm, including optimized hyperparameters

Depending on the training data and the (graph-)kernel parameters, features might be numerous and ineffectual. To save time in further processing, we directly drop some (very low cutoff) features based on random forest feature importance scores. Feature, model, and hyperparameter optimization is conducted via autoSKLearn [30], which applies efficient Bayesian optimization to find an effective model. autoSKLearn inspects 15 classification algorithms of the Python package scikit-learn and is capable to train ensemble models. Per default, all classification estimates are inspected for model selection. However, the list of estimators can be specified by the user.

### Model training using different data sources

CheRRI not only enables training based on interactomes from one organism but also allows to train a combined model for several organisms. To get a combined model, the training mode needs to be run for each individual organism first. In a subsequent training call, using the mixed-model mode, the different feature sets will be joined and a mixed model trained. The workflow is detailed in the online documentation linked above.

### Applying models on putative RRI sites

In CheRRI’s eval mode (see Fig. 1), a set of given putative RRI sites can be classified using a provided model. That is, their biological relevance is assessed based on the used model. Precomputed models for human and mouse can be downloaded from Zenodo [31]. An output table is computed providing the positions-based identifier of the given RRI sites and the predicted label (i.e., their final assessment of whether or not each site is expected to be biologically functional). It is recommended to also use the library of occupied regions that was generated and used for model training within the eval mode. That way, similar prediction constraints are applied. Furthermore, the same reference genome should be used.

### Data used to evaluate CheRRI

The training data were obtained from the DDD method PARIS [11] and further processed using the RNA–RNA interactome analysis tool ChiRA.

#### ChiRA data preparation

CheRRI’s training mode takes as input interaction summary files, which are one of the outputs of the ChiRA workflow. The input data of this study were extracted from the following Galaxy [32] history [33]. Novel data can also be prepared using ChiRA. A respective Galaxy tutorial is available [34].

#### Build of training instances

Out of the published PARIS data for the human HEK293T cell line and mouse embryonic stem cells dataset [11], 4 datasets were prepared in total: (i) “Human”, (ii) “Human + RBP” using RNA–protein interactome data [35], (iii) “Mouse”, and (iv) “Full” by merging the datasets (1 + 2 + 3). The RRI sites were filtered by a score of 1 to be considered “trusted”, meaning that only uniquely mapped reads are mapped to this interaction region. Only RRI sites found in all replicates were kept.

The “Human” and “Mouse” datasets were built using only RRI site information; that is, only RRIs are considered for the masking of occupied regions (see above). The “Human + RBP” dataset additionally incorporates regions involved in RNA–protein interactions as occupied. For the human HEK cell line, we used the RNA–protein interactome data from [35]. We extracted the crosslink positions of the protein occupancy cDNA libraries from SRA (GEO accession ID GSE38355) in the BedGraph format and converted the positions to the hg38 reference genome, as well as the BedGraph format to BED format. To capture more complete interaction sites, the crosslink positions were extended by 10 nt (i.e., 5 nt up- and downstream). For all datasets, trusted interaction regions were extended left and right by 150-nt genomic context (see Fig. 3). Using the occupied region information, a negative dataset was generated for evaluation as described earlier and in supplementary section S1.3. A summary of the dataset sizes can be found in Table 1.

**Table 1:** Dataset sizes of trusted RRI sites and their respective positive (pos) and negative (neg) RRI instances. The positive and negative instances are the up to 5 IntaRNA predictions per site.

		Training data	
Dataset	Trusted RRIs	Pos	Neg
Human	7,780	20,547	32,517
Human + RBP	7,780	14,845	27,744
Mouse	9,594	26,615	36,453

### Methods for model validation

CheRRI also provides the option to evaluate the predictive power of a model. To this end, positive and negative instances in the form of a validation dataset have to be provided. This can be done if the validation set is completely different from the train and test set (e.g., data from a different species) than the one of the model training. To cross-validate an individual model, a 5-fold cross-validation was used for the results of this publication. This functionality is part of the Auto-sklearn wrapper biofilm [36] that we integrated into CheRRI.

Example calls on how to perform the validation or cross-validation are given in CheRRI’s online documentation. The computation of the F1 score, the precision-recall curve (PRC), and the respective tables and plots were performed by scripts provided in CheRRI’s GitHub repository and can be used to reproduce the given results.

## Results and Discussion

### Model training

To train and evaluate CheRRI, the datasets described above were used to create 4 different models: (i) “human”, (ii) “human RBP”, (iii) “mouse”, and (iv) “full”, with the latter via merging the datasets (1 + 2 + 3). Note that datasets start with a capital letter and models with a lower letter. The model selection was performed on 5 cores allocating 9 GB per thread and giving the selection 18,000 s time. All final top-performing models use a histogram-based gradient boosting or a random forest classifier.

### Classification beyond thermodynamics

Most RRI prediction algorithms, like IntaRNA or TargetRNA [37], rely on an energy model to compute interactions, and thus the energy, as a measure of interaction stability, becomes the selection criterion for true interactions. To prune false-positive predictions from the top-scored interactions, we generate the negative training data for CheRRI on the bases of finding interaction sites that are not known to form an interaction but are still highly scored. This way, CheRRI is able to learn features that identify functional RRIs besides stability and avoid a strong bias toward interaction energy.

**Figure 4:**
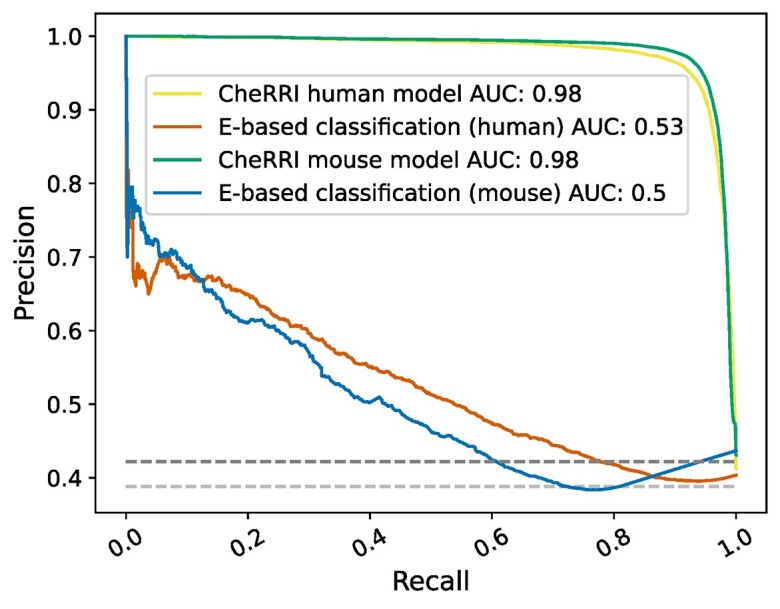
Precision-recall plot. Comparing CheRRI’s models for human and mouse (yellow and green line) against interaction site classification only based on minimal IntaRNA energy scores (E-based classification, orange [human] and blue line [mouse]). The dark gray line shows the baseline (e.g., how many predictions are expected to occur by chance) for the human data-based models and the lighter gray line for the mouse data-based models.

We assessed this hypothesis via a comparison of the predictive power of our models against a simple classification only based on minimal interaction energy per putative site reported by IntaRNA (E-based classification). Fig. 4 summarizes our findings for the Human and Mouse datasets in terms of PRCs. There is a clear distinction between the energy-based classification versus CheRRI’s models with additional features. As we can see, energy alone is not sufficient to distinguish functional from false-positive RRIs. The supplementary data show the PRCs for models with graph-kernel features in Supplementary Fig. S3 and the area under the curve (AUC) results in Supplementary Tables S7 and S9. The energy-based classification performs with 0.53 and 0.5 a bit better than random guessing (see gray baselines). The CheRRI models for both human and mouse have a very high detection rate (both 0.98 AUC).

Given the feature importance analysis detailed in the supplementary material, GC content and sequence complexity are among the most important features that distinguish the CheRRI model from the energy-only model, besides GC- and length-normalized features.

### Model comparison

**Figure 5:**
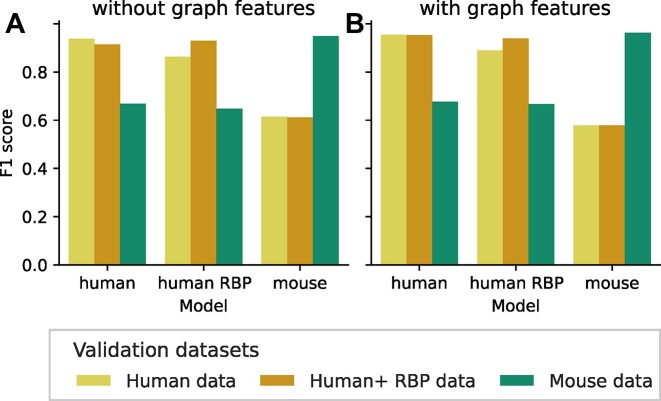
Evaluation of models. The model performance is measured by the F1 score, using different validation datasets. The figure compares the “Human”, “Human + RBP”, and “Mouse” datasets. On the left side (A) without graph-kernel models and (B) on the right side, including graph-kernel features. The model validation was performed using 5-fold cross-validation. All training data not used for a particular model training were used for validation (e.g., human model validated on mouse data).

Model performance (in terms of F1 score) was evaluated by 5-fold cross-validation or on datasets not used for training (see Supplementary Tables S6 and S8). The incorporation of graph-kernel features encoding the RRI base-pairing improved the F1 score of the cumulative model “full” from 0.94 (no graph-kernel features) to 0.95 (with graph-kernel features). The best performance was observed for the “human” model when applied to “Human + RBP” data incorporating RNA–protein interaction sites (0.95/0.91 F1 score with/without graph-kernel, respectively). Even evaluating “Mouse” interaction sites with the “human” model shows reasonable results (0.68/0.67 F1 score with/without graph-kernel, respectively). Using a “mouse” model for “Human” RRI evaluation still showed moderate F1 scores (0.59/0.61). The F1 comparisons can be seen in Fig. 5.

The “mouse” model can distinguish known interactions from the ones that are not present better than the “human” model (F1 score for human/mouse with and without graph-kernel: 0.95/0.96 and 0.94/0.95). The number of RRIs used for building training instances is higher for “mouse” (9,594) compared to “human” (7,780); see Table 1. However, applied to RRI sites of a different organism, the “human” model performs better.

When investigating the difference between human and mouse models in terms of feature importance (see supplementary material), we see strong importance differences for energy and GC-content features, while length-normalized variants show much lower deviations. This could result from a bias in one of the datasets toward specific classes of interactions or sequences and shows the known data sensitivity of machine learning–based models that can easily lead to overfitting. Thus, we conclude that revised datasets might reduce this limited portability of the tested organism-specific models. This hypothesis is also supported by the results of the “full” model that is trained on a combination of human and mouse data. This model shows excellent performance for all datasets in terms of F1 scores, which is shown in Supplementary Tables S5 and S7.

### Ad hoc usability via Galaxy

CheRRI is easily available via pip and conda package managers, via the bioconda channel [38], for command line usage and integration into data analysis workflows. To provide a graphical interface for an interactive user-friendly experience, CheRRI can also be found on Galaxy [32]. All results presented within the article are created via the Galaxy interface, which enables full reproducibility. All models trained on Galaxy are accessible via a Galaxy history [39]. CheRRI is publicly available on GitHub and can be used under a GPL-3.0 license [40].

## Conclusion

We introduce CheRRI, the first postprocessing pipeline to assess the biological relevance of putative RRI sites. Typically, RRI sites are assessed based on their minimum free energy, which is only a poor classifier. In contrast, CheRRI learns and applies highly accurate classification models that incorporate detailed features of RRIs and their context derived from reliable experimental interactome data. The latter should be filtered via RNA–RNA interactome evaluation pipelines like ChiRA or RNANUE [41] prior to using CheRRI to focus on highly reliable data.

CheRRI is easy to install (e.g., via conda) and flexible to use due to its built-in feature and model optimization. The Galaxy integration directly allows its application in reproducible workflows. Currently, CheRRI provides prebuilt models for human and mouse based on PARIS data (filtered using ChiRA). All models and feature sets are publicly available along with CheRRI and can be used to evaluate any given set of RRI sites.

Our analyses show that our models are still organism and data specific. This might result from an unknown bias within the data, the restriction to a specific experimental protocol, or the still limited training data size. The flexibility to extend the data and to curate and use own project-specific datasets is a central strength of CheRRI. Therefore, extended and revised datasets will continuously improve its reliability.

## Availability of Source Code and Requirements

- Project name: CheRRI
- Project homepage: https://github.com/BackofenLab/Cherri
- Operating system(s): CL-tool, Galaxy
- Programming language: Python
- License: GPL-3.0 license
- BioToolsID: biotools:cherri
- RRID: SCR_025175

An archival copy of the code is available via Software Heritage [42].

## Additional Files

**Supplementary Fig. S1**. Feature importance. Based on the human (yellow) and mouse (green) model data, the feature importance is calculated. The x-axis lists all hand-crafted features with corresponding feature importance scores on the y-axis.

**Supplementary Fig. S2**. Precision-recall curve, here comparing Minimum Free Energy (MFE) with graph feature models to the MFE base model for human and mouse. The dark gray line shows the baseline for the human data-based models and the lighter gray line for the mouse data-based models.

**Supplementary Fig. S3**. Benchmark workflow. Starting from a literature-based set of interacting RNA molecule sequences, 3 RNA–RNA interaction prediction tools (IntaRNA, RIsearch2, RIblast) were used to generate a list of putative RNA–RNA interaction sites. These were annotated whether they are correct or wrong based on localization information from literature. The putative RNA–RNA interaction sites were mapped to genomic coordinates to provide valid input for CheRRI. CheRRI’s classification of the sites was finally evaluated and compared to the respective correct/wrong categorization.

**Supplementary Table S1**. IntaRNA parameters set within CheRRI.

**Supplementary Table S2**. List of all hand-crafted (i.e., interaction and sequence) features.

**Supplementary Table S3**. MFE-based dataset size. The dataset is derived from the ChiRA PARIS data applied to CheRRI’s pipeline and taking only the best (lowest MFE) IntaRNA interaction prediction. Each column displays the number of data points originating from a different RRI original data source.

**Supplementary Table S4**. MFE-based model evaluation. Evaluation of how well the MFE separates between biologically relevant and nonrelevant (i.e., false-positive) interaction sites. Here the AUC of the precision-recall curve is calculated for the 3 datasets.

**Supplementary Table S5**. Evaluation of hand-crafted feature models (F1). The model performance is measured by the F1 score, using different evaluation/validation datasets. First-column names refer to the evaluation dataset and first-row names to the model that was used for the evaluation/validation.

**Supplementary Table S6**. Evaluation of hand-crafted feature models (AUC). The model performance is measured by the AUC of the precision-recall curve, using different evaluation/validation datasets. First-column names refer to the evaluation dataset and first-row names to the model that was used for the evaluation/validation.

**Supplementary Table S7**. Evaluation of models including additional graph-kernel features. The model performance is measured by the F1 score, using different evaluation/validation datasets. First-column names refer to the evaluation dataset and first-row names to the model that was used for the evaluation/validation.

**Supplementary Table S8**. Evaluation of models including additional graph-kernel features (AUC). The model performance is measured by the AUC of the precision-recall curve, using different evaluation/validation datasets. First-column names refer to the evaluation dataset and first-row names to the model that was used for the evaluation/validation.

**Supplementary Table S9**. Available data sources.

**Supplementary Table S10**. Annotation of predicted putative interaction sites based on their overlap with experimentally detected target regions.

**Supplementary Table S11**. Evaluation results for the benchmark dataset split by classes of interacting noncoding RNAs. For each class, the overall number of input sites (*n*) is given as well as the number of cases, where CheRRI provides no classification (NA). Classification results were compared with the literature-based “correct/wrong” annotation to count true-positive (TP), true-negative (TN), false-positive (FP), and false-negative (FN) classifications. Overall true and false classification counts are provided in the ∑ columns along with an F1 score.

## Abbreviations

AUC: area under the curve; DDD: direct duplex method; miRNA: microRNA; ML: machine learning; ncRNAs: noncoding RNAs; nt: nucleotides; PRC: precision-recall curve; RBP: RNA-binding protein; RRI: RNA–RNA interaction; sRNA: small regulatory RNA.

## Supplementary Material

giae022_supplement

giae022_GIGA_D_23_00295_Original_Submission

giae022_GIGA_D_23_00295_Revision_1

giae022_GIGA_D_23_00295_Revision_2

giae022_Response_to_Reviewer_Comments_Original_Submission

giae022_Response_to_Reviewer_Comments_Revision_1

giae022_Reviewer_1_Report_Original_Submission Tsukasa Fukunaga -- 11/15/2023

giae022_Reviewer_1_Report_Revision_1 Tsukasa Fukunaga -- 3/12/2024

giae022_Reviewer_2_Report_Original_Submission David Mathews -- 12/11/2023

giae021_Reviewer_2_Report_Revision_1

giae021_Reviewer_2_Report_Revision_2

## Data Availability

The generated models as well as data supporting the results of this article are available in the Zenodo repository [31].
