# Supplementary material for: CheRRI—Accurate classification of the biological relevance of putative RNA–RNA interaction sites
Source: Gigascience. 2024 Jun 5;13:giae022. doi: 10.1093/gigascience/giae022 (PMC11152173; doi:10.1093/gigascience/giae022)
Supplement: giae022_supplement [file giae022_supplement.zip › supplement/Cherri_GigaScience_final_submission_supplement.pdf]

CheRRI - Accurate classification of the biological relevance of putative RNA-RNA interaction sites Supplementary Material

Teresa Müller, Stefan Mautner, Pavankumar Videm, Florian Eggenhofer, Martin Raden, and Rolf Backofen

April 17, 2024

Contents

1 How CheRRI works **1**
- 1.1 RRI detection . . . 2
- 1.2 Formatting . . . 3
- 1.3 Occupied regions . . . 3
- 1.4 Context extension . . . 3
- 1.5 Interaction prediction . . . 4
- 1.6 Feature extraction . . . 4
- 1.7 Feature selection . . . 5
- 1.8 Model selection . . . 5
- 1.9 Classification . . . 7

2 Data preparation **7**
- 2.1 ChiRA data preparation . . . 7
- 2.2 CheRRI model data preparation . . . 7

3 Feature importance **8**

4 Model **9**
- 4.1 MFE-based RRI classification . . . 9
- 4.2 CheRRI model evaluation . . . 10

5 Data availability **10**

6 Benchmarking CheRRI **13**

1 How CheRRI works

In the following, we describe how CheRRI works. For full details on each individual parameter or usage of the tool, please check out CheRRI's online manual at https://backofenlab.github.io/Cherri/.

CheRRI can be run in two modes. The training mode (`cherri train`) creates an optimized classification model based on an RNA-RNA interactome (RNA-RNA interaction (RRI) site data).

The evaluation mode (`cherri eval`) takes in RRI position data given in a tabular format and reformats it, so it can be evaluated within the core CheRRI method. To run CheRRI evaluation, a reference genome and chromosome length file are needed as well since the instances are computed in the same way as they are computed for the model building. The full workflow of CheRRI can be seen in Figure 2 of the main paper, illustrating all input files in yellow, the method functions of CheRRI in blue, and the intermediate and final outputs in green. In the following, CheRRI's functions are described in more detail. As the figure highlights, both modes use the same core functions.

1.1 RRI detection

The inputs for the CheRRI model build step are the so-called 'interaction summary files' computed by the chimeric read analysis pipeline ChiRA [1]. For the model training, CheRRI only uses very reliable RRIs (the so-called trusted RRIs). CheRRI also makes use of replicates, by tracing and connecting RRI regions through all replicates using a region overlap cutoff.

Before computing an exact overlap, InterLap [2] is used to build a dictionary for a quick search for overlapping sequences in different replicates, where the keys contain strand and chromosome IDs, and the values are the interaction positions. Using this strategy to identify putative partners substantially decreases the runtime.

The overlaps between RRIs coming from different replicates R^1 and R^2 are calculated as follows: Each RRI has two interacting sequence regions $a = (s1, e1)$ and $b = (s2, e2)$ representing the start and end positions of the interacting sequences a and b. The exact overlap is calculated separately for both regions, i.e., between R_a^1 and R_a^2 and between R_b^1 and R_b^2. The overlapping sequences are computed by first finding the length of the overlapping region ($region$) via the genomic coordinates.

$$region_a = min(R_{e1}^1, R_{e1}^2) - max(R_{s1}^1, R_{s1}^2) + 1$$

Then the overlapping fraction for each of the RRI regions of the two replicates is calculated:

$$fractionR_a^1 = region_a/(R_{e1}^1 - R_{s1}^1 + 1)$$

$$fractionR_a^2 = region_a/(R_{e1}^2 - R_{s1}^2 + 1)$$

The maximum overlap of both replicate regions is taken as overlap fraction for the first sequence region ($overlap_a$).

$$overlap_a = max(fractionR_a^1, fractionR_a^2)$$

The overlap for the second sequence region (i.e., between R_b^1 and R_b^2) is calculated in the same way($overlap_b$). Then the average of the two regions is computed

$$overlap = (overlap_a + overlap_b)/2$$

and compared against the overlap threshold.

The RRI with the highest overlap in a replicate is picked as representative RRI and is later used to build the training dataset. Out of these, only RRIs with a score (i.e., the EM score computed by `ChiRA` and present in the `ChiRA` output files) of 1.0 are kept, which we refer to as trusted RRIs.

1.2 Formatting

The evaluation mode takes as input a tabular file containing position interaction information of a predicted RRI. This step converts the input table into the format needed by `CheRRI`'s core methods. So the features are generated in the same way as it was done for the model training. The same parameter settings (`IntaRNA` call, overlap and context) should be set as used for the provided model.

1.3 Occupied regions

Occupied regions are regions where a known interaction (i.e., found in the input data) is happening and is therefore unlikely part of another interaction. In order to compute negative instances, the set of occupied regions is utilized to find unknown (i.e., not found in the input data) but computationally possible interactions. The occupied regions are computed by using the set of given interactions. In training mode, these are all the traceable RRIs that are above an EM score of 0.5. The EM score computed by `ChiRA` defines the likelihood of an interaction to be uniquely mapped. A score of 1.0 indicates that the interaction is uniquely mapped. Multi-mapping results in lower scores. Hence, a score of 0.5 results in more occupied regions than the amount of trusted RRIs. For the evaluation mode, all given interacting regions are occupied regions. However, an additional precomputed occupied library can be specified by the user, e.g., the one used to build the model. The occupied regions are stored in an `InterLap` object (i.e., dictionary) to reduce the computation time. Additionally, the user can specify a BED file storing RNA-protein interaction crosslink positions, ideally from the same cell culture and experimental conditions, if available. For example, for our Human dataset, we utilized an RNA-protein interactome dataset for human embryonic kidney (HEK) cells from Baltz et al. [3].

1.4 Context extension

The genomic context extension is performed for two reasons. First, the context is needed to compute the accessibility of a given RRI by the prediction of its local structure. This allows the computation of the interaction hybrid and the extraction of additional features. Second, to have similar negative RRI hybrid interaction regions, we extract them in close neighborhood to the given interaction sites. `CheRRI` extracts a fixed context around the given interaction. The context sequence is added by using the fastaFromBed tool and the genomes in FASTA format. For human (hg38) and mouse (mm10), `CheRRI` automatically downloads the genomes from UCSC. For other species or genome versions, the user has to provide the genome file. Additionally, a chromosome length table file is needed.

We tested different context lengths and compared the feature set of positive and negative instances to each other (data not shown). As a result, we set the context length to 150 nt on both sides of the interaction. A shorter context leads to fewer negative instances, compared to positive ones. A longer context increases the amount of negative

instances, counteracting our desire to have negative interactions in close proximity to the positive ones. However, the optimal context length could vary for different datasets and is therefore adjustable.

1.5 Interaction prediction

In the next step the positive and negative interactions are computed using the state-of-the-art RRI prediction tool `IntaRNA` [4]. Due to its flexible usage, `IntaRNA` provides the platform to compute positive and negative instances. Figures 3 and 1 in the paper explain the data generation process. After the context extension, the occupied regions within the extended interaction regions are found. For the prediction of positive interactions, `IntaRNA` looks for a seed area (*seedT/QRang*) in the interaction region found by DDD methods. All other regions that are known to be involved in different interactions (i.e., occupied regions) are specified as regions that are not free for interaction (*q/tAccConstr*). For the prediction of the negative instances, the seed region is also defined as not free for interaction. `CheRRI` computes up to five best interactions for each instance to have an ensemble of interactions. Table S1 lists all relevant `IntaRNA` parameters and their values used inside `CheRRI`. All parameters that can be changed are defined in an external `IntaRNA` parameter file and can be added and changed by the user.

Table S1: `IntaRNA` parameters set within `CheRRI`.

parameter	value	description
seedBP	5	the number of base pairs within the seed
seedMinPu	0	the minimal unpaired probability of each seed region in query and target
accW	150	sliding window length (0=global folding)
accL	80	maximum length of considered intramolecular base pairs
acc	C	accessibility computation using the selected energy model for query or target sequences
outMaxE	-5	maximum energy for any interaction reported
outOverlap	B	overlapping of interaction sites of suboptimals allowed (B:both)
seedT/QRang	pos	genomic positions of the trusted RRI
q/tAccConstr	pos	genomic positions of the occupied regions
intLenMax	50	restrict the overall length of the interaction
temperature	37	experimental temperature (°C)
intLoopMax	3	number of unpaired bases between intermolecular base pairs
seedMaxE	0	threshold for the maximum seed energy

1.6 Feature extraction

`CheRRI` computes several features, in addition to those features pre-computed by `IntaRNA`. Both combined are referred to as hand-crafted features. Table S2 lists all hand-crafted (i.e., interaction and sequence) features. The pre-computed features by IntaRNA are the interaction energy E, E hybrid ('E_hybrind'), and the ED values ('ED'). The length of the interaction ('max_inter_len') is computed by the given coordinates of the IntaRNA interaction. The number of base pairs ('no_bp') is computed by counting the num-

ber of opening and closing base pairs of the predicted hybrid. The maximal interaction length will be initialized with the greater interaction length of either the target or the query interaction length. The interaction length normalized by the number of base pairs ('inter_len_normby_bp') is the maximal interaction length divided by the number of base pairs. The number of base pairs normalized by maximal interaction length ('bp_normby_inter_len').

The GC content ('GC_content') is computed by dividing the number of GC's contained in the interacting sequence by the length of the interacting sequence. The GC skew ('GC_skew') is again computed based on the interacting sequence by subtracting G from C divided by the sum of both. The AT skew ('AT_skew') is computed the same way [5]. The number of seeds ('no_seeds') is computed by counting the number of predicted seed start positions. The maximum ED value ('max_ED') is the maximum of the target and query ED value. There additional are the energy features, 'E', 'E_hybrind' and 'max_ED' which are normalized by 'max_inter_len', the maximal interaction length ('E_normby_len', 'E_hybrid_normby_len' and 'max_ED_normby_len'), and normalized by the 'GC_content' times the interaction length (E_normby_GC_len', 'E_hybrid_normby_GC_len' and 'max_ED_normby_GC_len').

The sequence complexity to the interacting sequence (complex_[target_query]_site) calculates the Shannon entropy according to the CE entropy calculation of Orlov and Potapov [6].

Further insight into the computation of the hand-crafted features can be gained in the get_features.py module of `CheRRI`.

In addition, `CheRRI` can use graph-kernel features calculated by EdeN [7]. Here a hybrid is modeled as a graph from which sub-graphs are explicitly turned into features using a hashing scheme similar to that of a Weisfeiler-Lehman kernel [8]. For the subgraph computation, the predicted interaction structure of IntaRNA is used.

1.7 Feature selection

Since the complexity of the additional graph-kernel features may violate memory constraints, we reduce the complexity of the training data by feature selection, before they are saved in a numpy NPZ file. Using the graph-kernel features the file './[output folder]/feature_file/training_[name]_train_context_[context].npz ' contains the already filtered feature and instance information. More specifically the compressed numpy file contains the data matrix, a label vector and a vector with feature identifiers. In both cases, the features are selected based on a random forest estimator, where an impurity-importance cutoff is found by fitting two Gaussians on the importance values and selecting the cutoff at the most ambiguous value.

This selection is reasonable since kernel-based feature generation is bound to produce many equally uninformative features. If no graph-kernel features are calculated, all hand-crafted features are stored in the feature file and directly applied to the `Auto-Sklearn` optimization pipeline.

1.8 Model selection

For the model selection and hyperparameter optimization, we use `Auto-Sklearn` [9]. For convenience, we built a thin wrapper around AutoSklearn (Biofilm - training FILtering

Table S2: List of all hand-crafted (i.e. interaction and sequence) features.

Feature	Description
E	Interaction energy computed by IntaRNA (E = E_hybrid + ED1 + ED2)
E_hybrid	Energy of hybridization only (E - ED1 - ED2)
ED1	Energy penalties for reduced accessibility of the target sequence
ED2	Energy penalties for reduced accessibility of the query sequence
max_ED	max(ED1, ED2)
sum_ED	ED1 + ED2
len_interaction_target	Length of the interacting target interaction site
len_interaction_query	Length of the interacting query interaction site
no_bps	Number of base pairs within the interaction
GC_content	GC-content of the interaction site
max_inter_len	The maximum interaction site length of target and query interaction site length
inter_len_normby_bp	The maximum interaction length divided by the number of base pairs within the interaction
bp_normby_inter_len	Number of base pairs within the interaction divided by the maximum length of the interaction
E_normby_GC	E divided by the GC_content
max_ED_normby_GC	max_ED divided by the GC_content
E_hybrid_normby_GC	E_hybrid divided by the GC_content
complex_target_site	Shannon entropy of interacting target subsequence
complex_query_site	Shannon entropy of interacting query subsequence
GC_skew	GC_skew = $(G - C)/(G + C)$
AT_skew	AT_skew = $(A - T)/(A + T)$
E_normby_len	Interaction energy E divided by the maximum interaction length
max_ED_normby_len	The max_ED divided by the maximum interaction length
E_hybrid_normby_len	E_hybrid divided by the maximum interaction length
E_normby_GC_len	E /(max_inter_len * GC_content)
max_ED_normby_GC_len	max_ED / (max_inter_len * GC_content)
E_hybrid_normby_GC_len	E_hybrid /(max_inter_len * GC_content)

Models for BIOlogical data). The wrapper is available on GitHub [10] and can be installed via conda. The number of jobs, the memory usage per job, and the optimization time should be adjusted to the given setup for an optimal `CheRRI` run.

1.9 Classification

The classification is part of `CheRRI`'s evaluation mode. Here a pre-trained model and its training data should be provided as input. The training data is needed to select the same features as used for the model training. Pre-trained models for human and mouse are provided (see Section 5 below). For the classification of RRI predictions from different organisms, we suggest that the user trains a model for this specific organism. After the classification, the output table will contain a column named 'predicted label', providing `CheRRI`'s prediction.

2 Data preparation

The training data was obtained from the DDD method PARIS [11] and further processed using the RNA-RNA interactome analysis tool `ChiRA`.

2.1 `ChiRA` data preparation

`CheRRI`'s training mode takes as input interaction summary files, which are one of the outputs of `ChiRA` workflow. The input data of this study was extracted from the following `Galaxy` [12] history: https://rna.usegalaxy.eu/u/videmp/h/paris-analysis Novel data can also be prepared by using `ChiRA`. A `Galaxy` tutorial on how to run `ChiRA` is available at: https://training.galaxyproject.org/training-material/topics/transcriptomics/tutorials/rna-interactome/tutorial.html.

2.2 `CheRRI` model data preparation

In total four datasets were prepared: (1) Human, (2) Human RBP using RNA-protein interactome data [3], (3) Mouse, and (4) Full merging the datasets (1+2+3). The RRI interaction regions were filtered by an EM score of 1 (trusted RRIs), meaning that only uniquely-mapped reads were mapped to this interaction region.

The Human and mouse datasets were built using only RRI site information to build the occupied regions dictionary. The Human RBP dataset additionally has the RNA-Protein interaction in the occupied regions dictionary. For the human HEK cell line, we used RNA-protein interactome data [3]. We extracted the crosslink positions of their protein occupancy cDNA libraries from SRA (GEO accession ID GSE38355) in BedGraph format, converted the positions to the hg38 reference genome, and the BedGraph format to BED format. To capture more complete interaction sites, the crosslink positions were extended by 10 nt (i.e., 5 nt up- and downstream). For all datasets trusted interaction regions were extended left and right by 150 nt. Using the occupied regions, negative interaction regions were generated as described in Section 1. A summary of the dataset sizes can be found in Table 1 of the main paper.

3 Feature importance

To check the feature importance we choose the RandomForestClassifier method from the sklearn library, using an impurity-based feature importance scoring. Therefore the higher the score the more important the feature is for final predictions. The scores are summarized in Fig. S1.

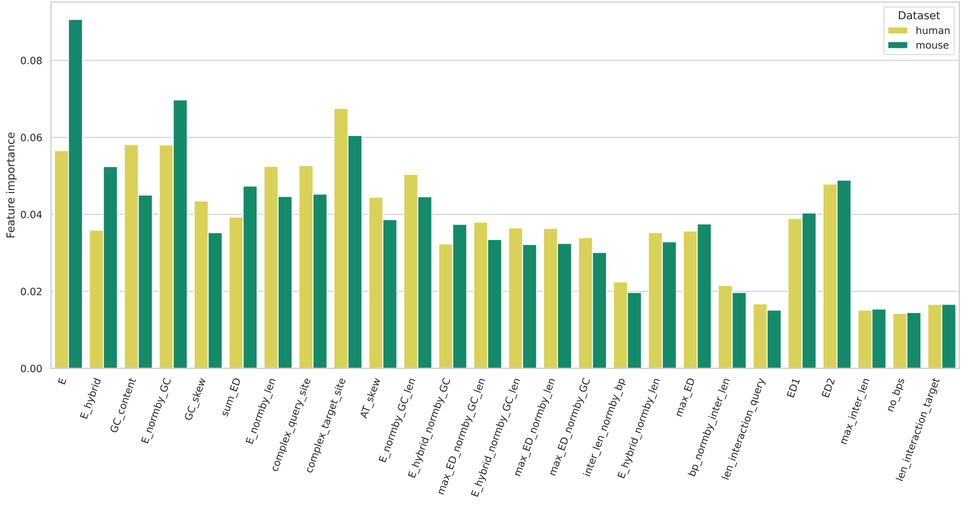

Figure S1: **Feature importance.** Based on the human (yellow) and mouse (green) model data the feature importance is calculated. The x-axis lists all hand-crafted features with corresponding feature importance scores on the y-axis.

The most important features besides the IntaRNA-predicted energy parameters E, E_hybrid and ED-values are the GC content, the sequence complexity and the normalization of the energy with the GC content. Interestingly, RRI length-encoding features like the number of base pairs (no_bp) and the interaction length for target and query have the lowest feature importance while their combination with other features for normalization (length-normalized features) are of much higher importance.

To understand which features are important for the different organism models it is interesting to look at the difference of the feature importance between the models. In Fig. S1 the features are sorted by the decreasing importance difference between the human and mouse model. The most important features show significant differences in their feature importance, between mouse and human, especially the energy values and the GC content. This could explain the difference between the two organism-specific trained models and their decrease in performance when the model is transferred to different organism data sources. If energy parameters are normalized by the interaction length the difference of importance between the models becomes smaller. However, also their overall importance is degrading. This hints at different energy and interaction length distributions in the provided data or the two organisms, which might be caused by a bias in the respective interaction data.

4 Model

From the positive and negative instances, the hand-crafted features, see Table S2, as well as graph-kernel features, are extracted. Models, based on these features and optimally produced by the wrapper `biofilm`, can be used as a root for post-processing prediction or experimental RRI site data.

4.1 MFE-based RRI classification

To our knowledge, there exists no method like ours with the ability to post-filter predictions based on the biological relevance of the interaction site. In theory, users of a prediction method like `IntaRNA` would use the minimum free energy (MFE) to search for biologically relevant interactions. Therefore `CheRRI` data processing was run using only the prediction having the lowest MFE (default five lowest MFE predictions). The number of data points are lower, see Table S3, than the one used for model training, see Table 1 main paper.

Table S3: **MFE based dataset size** The dataset is derived from the `ChiRA` Paris data applied to `CheRRI`s pipeline and taking only the best (lowest MFE) `IntaRNA` interaction prediction. Each column displays the number of data points originating from a different RRI original data source.

Dataset	Human	Human RBP	Mouse
Positive instances	4504	3242	5815
Negative instances	6657	5718	7497

We estimated the biological relevance detection power of MFE with the area under the curve (AUC) of the precision-recall curve for our three datasets Table S4 using the original data. These results suggest, that it is not recommendable to only use the MFE to evaluate the biological relevance of a predicted interaction site. To better interpret the results a baseline is given, helping to asses how many positive predictions are expected to occur by chance, baseline = positive datapoints/(positive datapoints + negative datapoints).

Table S4: **MFE base model evaluation.** Evaluation of how well the MFE separates between biologically relevant and non-relevant (i.e., false positive) interaction sites. Here the area under the curve (AUC) of the precision-recall curve is calculated for the three datasets.

Dataset	Human	Human RBP	Mouse
AUC hand-crafted features	0.49	0.42	0.46
AUC with graph features	0.49	0.42	0.46

Further, we plotted the PRC for the human and mouse model against an MFE comparison, but here only taking the single best MFE of the interaction and not the top 5. Therefore, there are fewer data points in the MFE comparison than for the models (still using 5 suboptimal interactions). Figure 3 of the main paper shows the PRC for the hand-crafted models and Figure S2 for the graph-feature models.

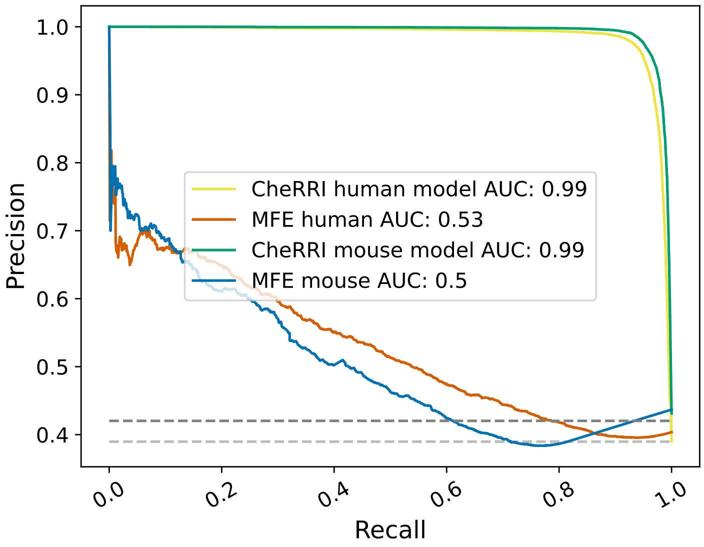

Figure S2: **Precision-Recall curve** Here comparing MFE with graph features Models to the MFE base model for human and mouse. The dark gray line shows the baseline for the human data-based models and the lighter gray line for the mouse data-based models.

4.2 `CheRRI` model evaluation

Starting from the three datasets, in total eight optimized models were trained. The first three models are based solely on the hand-crafted features (Human, Human RBP, Mouse). In addition, a full model based on the combination of the three datasets was trained from the hand-crafted features. Likewise, four models were trained including the graph features (Human, Human RBP, Mouse, Full).

The models were evaluated on all datasets (including the datasets from the other models), using the F1 score as a quality metric. The performance of the models was evaluated by 5-fold cross-validation on their respective training datasets, i.e., by splitting the data into 5 parts. Results can be seen in Table S5 for the hand-crafted features only and in Table S7 for the models trained with graph-kernel features. Additionally using the predictive probability the AUC was computed in the same way as for the MFE base model evaluation. These results are summarized in Table S6 for the hand-crafted features only and in Table S8 for the models trained with graph-kernel features.

5 Data availability

The RNA-RNA interactome, which is analyzed by `ChiRA`, is available via the `Galaxy` history. The original PARIS data is discussed in [1]. All trained models described in this publication can be downloaded from Zenodo. Table S9 summarizes all the available data.

Table S5: **Evaluation of hand-crafted feature models (F1).** The model performance is measured by the F1 score, using different evaluation/validation datasets. First-column names refer to the evaluation dataset, and first-row names to the model which was used for the evaluation/validation.

Model/ Dataset	full	human	human rbp	mouse
Full	0.94	0.84	0.81	0.78
Human	0.99	0.94	0.86	0.61
Human + RBP	0.99	0.91	0.93	0.61
Mouse	0.99	0.67	0.65	0.95

Table S6: **Evaluation of hand-crafted feature models (AUC).** The model performance is measured by the area under the curve (AUC) of the precision-recall curve, using different evaluation/validation datasets. First-column names refer to the evaluation dataset, and first-row names to the model which was used for the evaluation/validation.

Model/ Dataset	Full	Human	human rbp	Mouse
Full	0.98	0.92	0.90	0.88
Human	1.00	0.98	0.94	0.69
Human + RBP	1.00	0.97	0.98	0.67
Mouse	1.00	0.76	0.76	0.98

Table S7: **Evaluation of models including additional graph-kernel features.** The model performance is measured by the F1 score, using different evaluation/validation datasets. First-column names refer to the evaluation dataset, and first-row names to the model which was used for the evaluation/validation.

Model/ Dataset	full	human	human rbp	mouse
Full	0.95	0.86	0.83	0.77
Human	1.00	0.95	0.89	0.58
Human + RBP	1.00	0.95	0.94	0.58
Mouse	0.99	0.68	0.67	0.96

Table S8: **Evaluation of models including additional graph-kernel features (AUC).** The model performance is measured by the area under the curve (AUC) of the precision-recall curve, using different evaluation/validation datasets. First-column names refer to the evaluation dataset, and first-row names to the model which was used for the evaluation/validation.

Model/ Dataset	full	human	human rbp	mouse
Full	0.99	0.95	0.93	0.88
Human	1.00	0.99	0.96	0.69
Human + RBP	1.00	0.99	0.98	0.67
Mouse	1.00	0.79	0.80	0.99

Table S9: Available data sources.

Dataset	Download location
PARIS RRI data	https://rna.usegalaxy.eu/u/videmp/h/paris-analysis
Models	Zenodo DOI: https://doi.org/10.5281/zenodo.6533931
Human genome	https://hgdownload.cse.ucsc.edu/goldenpath/hg38/bigZips/hg38.fa.gz
Human length file	https://hgdownload.cse.ucsc.edu/goldenpath/hg38/bigZips/hg38.chrom.sizes
Mouse genome	https://hgdownload.soe.ucsc.edu/goldenPath/mm10/bigZips/mm10.fa.gz
Mouse length file	https://hgdownload.soe.ucsc.edu/goldenpath/mm10/bigZips/mm10.chrom.sizes
RNA-Protein interactome for HEK cells	GSE38355

6 Benchmarking CheRRI

CheRRI is designed as a filter to reduce false positive data from genome wide interaction information. In order to benchmark CheRRI, a data set of experimentally verified location information of interactions is needed in order to assess whether CheRRI's classification is in line with experimental evidence or not. Most reliable sources are mutation experiments that verify via substitutions or deletions that the mutated subsequence (covering the core of a hypothesized RNA-RNA interaction) is indeed needed to cause a detectable effect, which typically implies and proves its involvement in the hypothesized RNA-RNA interaction of interest.

Unfortunately, to the best of our knowledge, no such data set exists for human and mouse, for which CheRRI was trained in this study. Available data bases like RNAInter [13] or NPInter [14] are only providing interaction information in terms of "are pairing" without interaction details or specific location information, since they are mainly tailored to study interaction networks.

The only data partially in line with our requirements is provided by Umu and Gardener [4], who aggregated a benchmark data set to compare different RNA-RNA interaction prediction tools. We extracted from the benchmark data set all 38 human interaction partners present, which covers 15 snoRNAs, 21 miRNAs, and 2 snRNAs with their respective target RNAs. Note, the data set provides final transcription products. Furthermore, only for the target RNAs interaction localization information is available. Given this information, we designed a benchmark workflow as follows to evaluate CheRRI, which is depicted in Fig. S3.

In short, we used RNA-RNA interaction prediction tools (IntaRNA [15], RIsearch2 [16], and RIblast [17]) to generate putative interaction sites for experimentally studied RNA-RNA interactions to be classified by CheRRI. Since not all tools provide predictions for all sequence pairs, we end up with 75 putative interactions (IntaRNA 37, RIsearch2 21, RIblast 17). In order to be able to assess whether CheRRI's classification is true, we check whether the putative interaction sites overlap within the target molecules with the experimentally determined location information from the benchmark data to annotate them as correct or wrong, which is summarized in Tab. S10.

Tool	Overall	Correct	Wrong
IntaRNA	37	20	17
RIsearch2	21	12	9
Riblast	17	9	8

Table S10: Annotation of predicted putative interaction sites based on their overlap with experimentally detected target regions.

To evaluate the correctness of CheRRI's classification of all correct and wrong putative interaction sites, we applied the human model including protein binding sites without graph features. Table 6 presents the results.

miRNAs are most abundant (34) in the data set and classified with the highest number of correct classifications (20) and an F1 score of 0.61. Two classes of snoRNA interactions are present. For both CheRRI predictions were less in line with the literature-based annotation (only 15 of 39). For both, miRNAs and snoRNAs, CheRRI was not able to classify the interaction site for 10 sites in total.

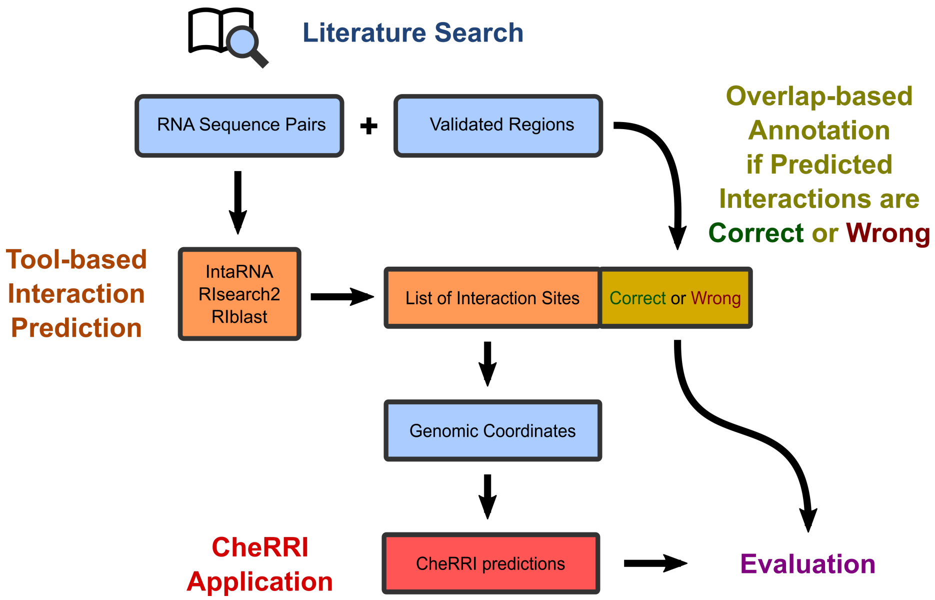

Figure S3: Benchmark workflow. Starting from a literature-based set of interacting RNA molecule sequences, three RNA-RNA interaction prediction tools (IntaRNA, RIsearch2, RIblast) were used to generate a list of putative RNA-RNA interaction sites. These were annotated whether they are correct or wrong based on localization information from literature. The putative RNA-RNA interaction sites were mapped to genomic coordinates to provide valid input for `CheRRI`. `CheRRI`'s classification of the sites was finally evaluated and compared to the respective correct/wrong categorization.

Overall, `CheRRI` is mainly correct and conservative in its classification, which manifests in high overall true classifications and higher FN than FP rates. But the differences for the individual RNA classes hint already at the limitations of the benchmark. While the benchmark workflow fills the gap of the missing gold standard benchmark data, it has various intrinsic problems. Most important, the data is biased due to its size and since only three types of RNA classes are represented, where both miRNAs and snoRNAs pose individual problems.

In detail, snoRNAs are known to form multiple concurrent subinteractions to fulfill their regulatory function but (i) all used tools are only able to predict one single consecutive interaction site and (ii) it is unclear if all interacting target regions are covered by the annotated localization information of the benchmark data from [4].

Furthermore, miRNAs are short non-coding RNAs that are post-transcriptionally extracted from a fully complementary hairpin-loop-forming precursor molecule. While the tools used only the mature miRNA molecule to predict the putative interaction sites, `CheRRI` extracts the site (i.e. miRNA sequence) with its genomic context for classification. Thus, interaction features of `CheRRI` are based on a sequence more related to the precursor that is able to form the hairpin loop, which strongly influences accessibility and thus interaction features. Finally, some of the target RNAs are rRNAs, which define and stabilize their structure by other molecules that are not modeled.

RNA class	n	NA	TP	TN	FP	FN	$\sum$ T*	$\sum$ F*	F1
miRNA	34	3	11	9	4	7	20	11	0.61
C/D snoRNA	21	3	7	2	3	6	9	9	0
H/ACA snoRNA	18	4	0	6	3	5	6	8	0.67
snRNA	2	0	2	0	0	0	2	0	1
overall	75	10	20	17	10	18	37	28	0.59

Table S11: Evaluation results for the benchmark data set split by classes of interacting non-coding RNAs. For each class, the overall number of input sites (n) is given as well as the number of cases, where `CheRRI` provides no classification (NA). Classification results were compared with the literature-based "correct/wrong" annotation to count true positive (TP), true negative (TN), false positive (FP), and false negative (FN) classifications. Overall true and false classification counts are provided in the $\sum$ columns along with an F1 score.

Thus we conclude that the compilation of a representative and sound gold standard data set to benchmark eukaryotic RNA-RNA interaction prediction and classification tools that operate on a genomic level and provides experimentally validated localization information is an open challenge well beyond the scope of this work.

References

[1] Pavankumar Videm, Anup Kumar, Oleg Zharkov, Björn Andreas Grüning, and Rolf Backofen. Chira: an integrated framework for chimeric read analysis from RNA-RNA interactome and RNA structurome data. *GigaScience*, 10(2):giaa158, 2021.

[2] Brent Pedersen. InterLap: simple, fast interval overlap testing. *GitHub repository*, 2014. https://github.com/brentp/interlap.

[3] Alexander G Baltz, Mathias Munschauer, Björn Schwanhäusser, Alexandra Vasile, Yasuhiro Murakawa, Markus Schueler, Noah Youngs, Duncan Penfold-Brown, Kevin Drew, Miha Milek, et al. The mRNA-bound proteome and its global occupancy profile on protein-coding transcripts. *Molecular cell*, 46(5):674–690, 2012.

[4] Sinan Uğur Umu and Paul P Gardner. A comprehensive benchmark of RNA–RNA interaction prediction tools for all domains of life. *Bioinformatics*, 33(7):988–996, 2017.

[5] Andrei Grigoriev. Analyzing genomes with cumulative skew diagrams. *Nucleic acids research*, 26(10):2286–2290, 1998.

[6] Yuri L Orlov and Vladimir N Potapov. Complexity: an internet resource for analysis of dna sequence complexity. *Nucleic acids research*, 32(suppl_2):W628–W633, 2004.

[7] Nicolò Navarin and Fabrizio Costa. An efficient graph kernel method for non-coding RNA functional prediction. *Bioinformatics*, 33(17):2642–2650, 05 2017.

[8] Nino Shervashidze, Pascal Schweitzer, Erik Jan Van Leeuwen, Kurt Mehlhorn, and Karsten M Borgwardt. Weisfeiler-lehman graph kernels. *Journal of Machine Learning Research*, 12(9), 2011.

[9] Matthias Feurer, Aaron Klein, Jost Eggensperger, Katharina Springenberg, Manuel Blum, and Frank Hutter. Efficient and Robust Automated Machine Learning. In *Advances in Neural Information Processing Systems 28 (2015)*, pages 2962–2970, 2015.

[10] Stefan Mautner. Biofilm. *GitHub repository*, 2021. https://github.com/smautner/biofilm.

[11] Zhipeng Lu, Qiangfeng Cliff Zhang, Byron Lee, Ryan A Flynn, Martin A Smith, James T Robinson, Chen Davidovich, Anne R Gooding, Karen J Goodrich, John S Mattick, et al. RNA duplex map in living cells reveals higher-order transcriptome structure. *Cell*, 165(5):1267–1279, 2016.

[12] Enis Afgan, Dannon Baker, Bérénice Batut, Marius Van Den Beek, Dave Bouvier, Martin Čech, John Chilton, Dave Clements, Nate Coraor, Björn A Grüning, et al. The Galaxy platform for accessible, reproducible and collaborative biomedical analyses: 2018 update. *Nucleic acids research*, 46(W1):W537–W544, 2018.

[13] Yunqing Lin, Tianyuan Liu, Tianyu Cui, Zhao Wang, Yuncong Zhang, Puwen Tan, Yan Huang, Jia Yu, and Dong Wang. RNAInter in 2020: RNA interactome repository with increased coverage and annotation. *Nucleic Acids Research*, 48:D189–D197, 2020.

[14] Yu Zheng, Huaxia Luo, Xueyi Teng, Xinpei Hao, Xiaoyu Yan, Yiheng Tang, Wanyu Zhang, Yuanxin Wang, Peng Zhang, Yanyan Li, Yi Zhao, Runsheng Chen, and Shunmin he. NPInter v5.0: ncRNA interaction database in a new era. *Nucleic acids research*, 51:D232–D239, 2023.

[15] Martin Mann, Patrick R. Wright, and Rolf Backofen. IntaRNA 2.0: enhanced and customizable prediction of RNA-RNA interactions. 45(W1):W435–W439, 2017.

[16] Ferhat Alkan, Anne Wenzel, Oana Palasca, Peter Kerpedjiev, Anders Rudebeck, Peter Stadler, Ivo Hofacker, and Jan Gorodkin. RIsearch2: suffix array-based large-scale prediction of RNA-RNA interactions and siRNA off-targets. *Nucleic acids research*, 45:e60, 2017.

[17] Tsukasa Fukunaga and Michiaki Hamada. Riblast: An ultrafast rna-rna interaction prediction system based on a seed-and-extension approach. *Bioinformatics (Oxford, England)*, 33:2666–2674, 2017.
