# Supplementary figures and images for: CheRRI—Accurate classification of the biological relevance of putative RNA–RNA interaction sites
Source: Gigascience. 2024 Jun 5;13:giae022. doi: 10.1093/gigascience/giae022 (PMC11152173; doi:10.1093/gigascience/giae022)

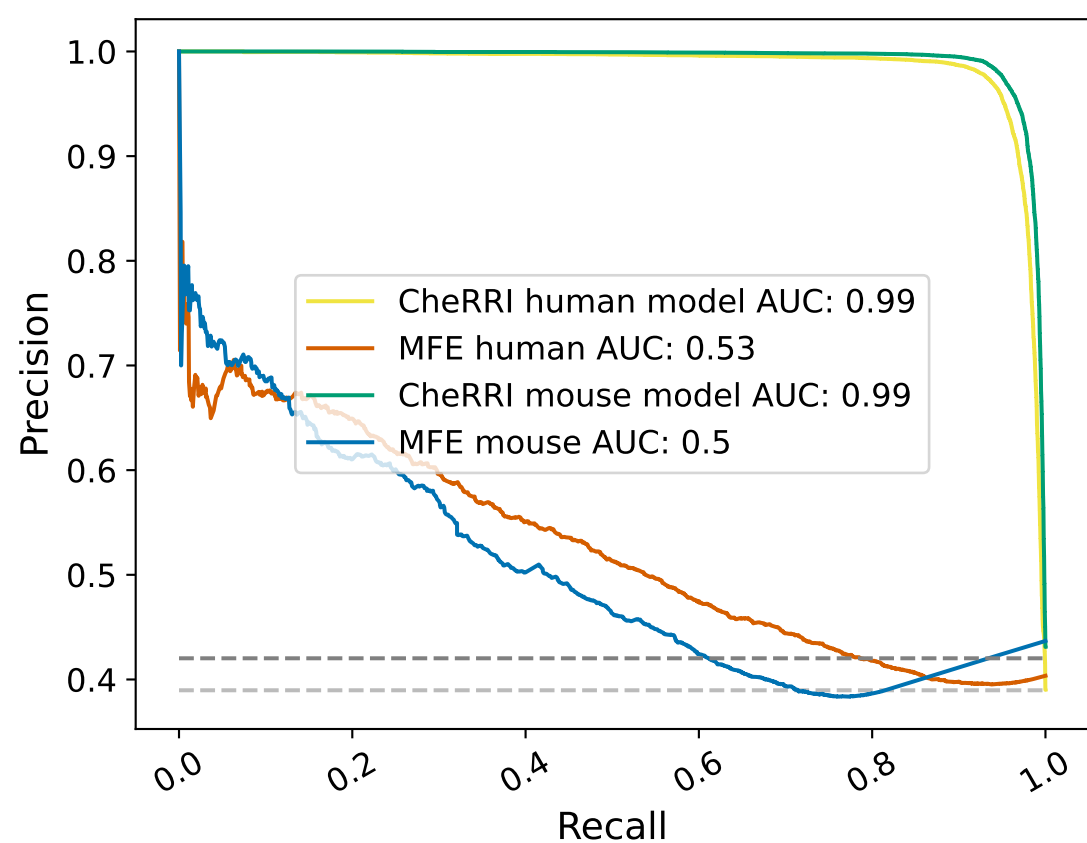

Supplement: giae022_supplement [file giae022_supplement.zip › supplement/prc_plot_supp.pdf]
