# Supplementary material for: CheRRI—Accurate classification of the biological relevance of putative RNA–RNA interaction sites
Source: Gigascience. 2024 Jun 5;13:giae022. doi: 10.1093/gigascience/giae022 (PMC11152173; doi:10.1093/gigascience/giae022)
Supplement: giae022_GIGA_D_23_00295_Revision_2 [file giae022_giga_d_23_00295_revision_2.pdf]

GigaScience

CheRRI - Accurate classification of the biological relevance of putative RNA-RNA interaction sites.

--Manuscript Draft--

**Manuscript Number:**	GIGA-D-23-00295R2
**Full Title:**	CheRRI - Accurate classification of the biological relevance of putative RNA-RNA interaction sites.
**Article Type:**	Technical Note
**Funding Information:**	Bundesministerium für Bildung und Forschung (RNAProNet-031L0164B) — Mr. Rolf Backofen<br>Deutsche Forschungsgemeinschaft (SFB 1425/1) — Mr. Rolf Backofen<br>Deutsche Forschungsgemeinschaft (390939984) — Mr. Rolf Backofen<br>Deutsche Forschungsgemeinschaft (EXC-2189) — Mr. Rolf Backofen
**Abstract:**	Background: RNA-RNA interactions are key to a wide range of cellular functions. The detection of potential interactions helps to understand the underlying processes. However, potential interactions identified via in silico or experimental high throughput methods can lack precision because of a high false positive rate.<br>Results: We present CheRRI, the first tool to evaluate the biological relevance of putative RNA-RNA interaction sites. CheRRI filters candidates via a machine learning-based model trained on experimental RNA-RNA interactome data. Its unique setup combines interactome data and an established thermodynamic prediction tool to integrate experimental data with state-of-the-art computational models. Applying this data to an automated machine-learning approach provides the opportunity to not only filter data for potential false positives but also to tailor the underlying interaction site model to specific needs.<br>Conclusion: CheRRI is a stand-alone postprocessing tool to filter either predicted or experimentally identified potential RNA-RNA interactions on a genomic level to enhance the quality of interaction candidates. It is easy to install (via conda, pip packages), use (via Galaxy) and integrate into existing RNA-RNA interaction pipelines.
**Corresponding Author:**	Teresa Müller<br>University of Freiburg: Albert-Ludwigs-Universitat Freiburg<br>Freiburg, GERMANY
**Corresponding Author Secondary Information:**	
**Corresponding Author's Institution:**	University of Freiburg: Albert-Ludwigs-Universitat Freiburg
**Corresponding Author's Secondary Institution:**	
**First Author:**	Teresa Müller
**First Author Secondary Information:**	
**Order of Authors:**	Teresa Müller<br>Stefan Mautner<br>Pavankumar Videm, Dr.<br>Florian Eggenhofer, PhD<br>Martin Raden, Dr.<br>Rolf Backofen, Dr.
**Order of Authors Secondary Information:**	

Powered by Editorial Manager® and ProduXion Manager® from Aries Systems Corporation

Response to Reviewers:	Dear Hans,<br><br>Thank you again for accepting and processing our publication. We addressed all of your formatting and editorial points.<br><br>Best wishes,<br>Teresa
Additional Information:	
Question	Response
Are you submitting this manuscript to a special series or article collection?	No
**Experimental design and statistics**<br><br>Full details of the experimental design and statistical methods used should be given in the Methods section, as detailed in our Minimum Standards Reporting Checklist. Information essential to interpreting the data presented should be made available in the figure legends.<br><br>Have you included all the information requested in your manuscript?	Yes
**Resources**<br><br>A description of all resources used, including antibodies, cell lines, animals and software tools, with enough information to allow them to be uniquely identified, should be included in the Methods section. Authors are strongly encouraged to cite Research Resource Identifiers (RRIDs) for antibodies, model organisms and tools, where possible.<br><br>Have you included the information requested as detailed in our Minimum Standards Reporting Checklist?	Yes
**Availability of data and materials**<br><br>All datasets and code on which the conclusions of the paper rely must be either included in your submission or deposited in publicly available repositories	Yes

(where available and ethically appropriate), referencing such data using a unique identifier in the references and in the “Availability of Data and Materials” section of your manuscript.<br><br>Have you have met the above requirement as detailed in our Minimum Standards Reporting Checklist?	

Powered by Editorial Manager® and ProduXion Manager® from Aries Systems Corporation

Main paper
Click here to access/download;Manuscript;Cherri_GigaScience_final_submi

OXFORD (GIGA)^n SCIENCE

GigaScience, 2017, 1–7

doi: xx.xxxx/xxxx
Manuscript in Preparation
technical note

TECHNICAL NOTE

CheRRI - Accurate classification of the biological relevance of putative RNA-RNA interaction sites

Teresa Müller [1,*], Stefan Mautner [1], Pavankumar Videm [1], Florian Eggenhofer [1,†], Martin Raden [1,†] and Rolf Backofen [1,2,*,†]

[1]Bioinformatics Group, Department of Computer Science, University of Freiburg, Georges-Koehler-Allee 106, 79110 Freiburg, Germany and [2]Signalling Research Centres BIOSS and CIBSS, University of Freiburg, Schaenzlestr. 18, 79104 Freiburg, Germany

[*]muellert@informatik.uni-freiburg.de; backofen@informatik.uni-freiburg.de
[†]Contributed equally.

Abstract

Background: RNA-RNA interactions are key to a wide range of cellular functions. The detection of potential interactions helps to understand the underlying processes. However, potential interactions identified via in silico or experimental high throughput methods can lack precision because of a high false positive rate.
Results: We present CheRRI, the first tool to evaluate the biological relevance of putative RNA-RNA interaction sites. CheRRI filters candidates via a machine learning-based model trained on experimental RNA-RNA interactome data. Its unique setup combines interactome data and an established thermodynamic prediction tool to integrate experimental data with state-of-the-art computational models. Applying this data to an automated machine-learning approach provides the opportunity to not only filter data for potential false positives but also to tailor the underlying interaction site model to specific needs.
Conclusion: CheRRI is a stand-alone postprocessing tool to filter either predicted or experimentally identified potential RNA-RNA interactions on a genomic level to enhance the quality of interaction candidates. It is easy to install (via conda, pip packages), use (via Galaxy) and integrate into existing RNA-RNA interaction pipelines.

Key words: RNA-RNA interactome; direct duplex detection; classification; functional RRI; false positives

Background

RNA-RNA interactions (RRIs) are fundamental for many cellular processes [1]. Non-coding RNAs (ncRNAs) regulate gene expressions on a transcriptional level as well as post-transcriptional, like long non-coding RNAs [2] or miRNAs [3]. Bacterial small regulatory RNAs (sRNAs) regulate all kinds of cellular processes and are therefore studied on a genome-wide scale [4, 5]. Some ncRNAs are parts of complex regulation networks, which complicates their investigation [6].

The importance of RNA-RNA interactions has given rise to RNA-interactome databases like RISE [7] or RNAInter [8], providing immense datasets of interactions. These datasets, however, show a high diversity in terms of data source, quality, and reliability. Gong et al. [7] discussed that the overlap between RRIs is varying between different experimental protocols, and also between different prediction methods.

A more unified picture of the RNA-interactome of a specific organism is provided by Direct Duplex Detection (DDD) methods. They are based on high-throughput crosslinking structure analysis without using specific RNA-binding proteins and are a promising source of reliable transcriptome-wide experimental RRI data [9, 10]. Different protocols like PARIS [11], LIGR-seq [12], and SPLASH [13] are available, out of which PARIS was found to identify the highest number of RRI sites [14]. An RRI site, as identified by a DDD method, is defined by two crosslinked (short) subsequences but does not

Compiled on: April 17, 2024.
Draft manuscript prepared by the author.

Key Points

- Classification of putative RNA-RNA interaction (RRI) sites concerning their biological relevance
- Filtering of false positive RRIs
- Automated model building for custom interactome data

provide details about the occurring intermolecular base pairing. Since crosslinking is not always caused by true RRIs, DDD results are also listing false positive RRI sites.

Another commonly used approach for detecting putative RNA-RNA interactions is the computational prediction of potential intermolecular base pairs. Typically, programs like `RIblast` [15], `RIsearch2` [16], or `IntaRNA` [17] are used, while the latter was found to be among the most reliable of the available bioinformatics tools [18, 19] with flexibly adjustable constraints [20]. There are recent efforts to solve RRI predictions using machine learning methods [21, 22, 23]. These approaches are, however, typically tailored to a specific type of regulatory RNAs like miRNAs [24] or prokaryotic sRNAs [25]. To face the high amount of putative predictions and high false positive rates, such methods often combine prediction with feature-based filtering, e.g. for the selection of potent siRNAs [26]. While such filters are effective, they are currently tailored to and integrated into respective tools and cannot be applied on putative RRI sites derived from other sources.

So far, there exists no method-independent approach to filter already identified potential RRI prediction sites based on their biological relevance; where relevance should be based on distinguishing features learned from a curated dataset that is based on experimentally identified RNA-RNA interactome data. Such a filtering method would allow to post-process experimental data to prune false positives resulting from wrong predictions or protocol artifacts.

Here, we introduce `CheRRI` to fill this gap. To enable the creation of a reliable model, the `CheRRI` curation sub-pipeline applies several automated quality checks to extract from a given potential training data set a subset of reliable RRI sites. Based on the reliable RRI sites, `CheRRI` derives features from interactome-constraint RRI predictions, as well as additional sequence, context, and graph features. For instance, also information about known binding sites of RNA-binding proteins (RBPs) can be incorporated into the model, since RBPs are known to influence the local structure of RNAs and thus their binding capacity [27]. The generated features are used to automatically learn the best classification model for a given training dataset and to provide a pre-computed, high-quality RRI evaluation model. An overview of the approach is given in Figure 1. Our `CheRRI` tool provides, after learning, an evaluation mode that allows to filter putative RRI sites. That way, we can remove false positive RRI sites from data produced by both experimental or in-silico methods.

Approach

`CheRRI` uses genome-wide experimental RNA-RNA interactome data to train a classification model for a subsequent evaluation of putative RRI sites, see Figure 1. `CheRRI` provides pre-trained human (HEK293T cell line) and mouse (embryonic stem cells) models for its direct application to filter user-provided potential RRI sites in both organisms. To this end, the user only has to provide a set of putative RRI sites, i.e., genomic positions of both interacting subsequences. If not interested in the built-in human and mouse genomes, the corresponding reference genome is to be given as well. `CheRRI` is a pipeline built of different submodules and functions that are depicted in Figure 2. A detailed functionality description is given in section S1 of the supplementary material and in the online documentation https://backofenlab.github.io/Cherri/.

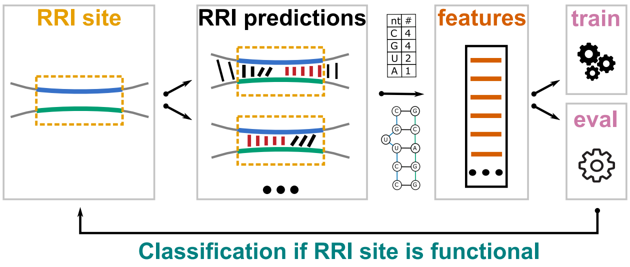

Figure 1. Graphical abstract `CheRRI` takes RRI sites (yellow) as input and adds genomic context up- and downstream (grey). Inside these extended RRI sites, RRI predictions (black, red) are computed by `IntaRNA`. The RRI prediction can exceed the original RRI site (fine black), but the seed (red) needs to be within the RRI site. `CheRRI` then extracts various sequence, context, and graph features (orange). These features are used to train a predictive model (`train` mode), or to evaluate whether a given RRI site is biologically relevant (`eval` mode).

To train a new evaluation model with `CheRRI`, a processed DDD dataset has to be provided. More precisely, it consists of sets of reliable RRI sites (i.e., pairs of short subsequences that were found to be interacting) in different replicates and, if possible, in concert with additional information like protein binding sites. Thus, `CheRRI` is not restricted to a specific HTS interactome protocol or a dedicated chimeric read analysis pipeline. It solely needs the RRI site information. Details about data preparation, model training, and application are provided in the following sections.

Preparing training via extraction of reliable RRI sites from interactome data

Our RRI site classification model is based on experimental RNA-RNA interactome data. In order to generate a reliable model, the provided data needs to be pruned to "trustworthy" RRIs. This is done based on replicate and read analyses.

In case replicates are provided, only interactions present in all replicates are kept for further filtering and model generation. This is detected via an overlap comparison of the RRI sites. Boundary accuracy of detected RRI sites from different protocols can vary. Thus, based on preliminary analyses, we set the default overlap threshold to 30%. Technical details are provided in section S1.1 of the supplementary material.

To further enhance the data set, we subsequently filter for reliable RRI sites via their EM scores, which can be computed with the chimeric read analyzer `ChiRA` [28]. We use a conservative default cutoff for the EM score (≥ 1), since we want to keep only sites that are highly reliable.

In summary, via the use of two filtering criteria, i.e. (1) detection of RRI sites shared among replicates and (2) the selection of high-quality mapped reads, it is ensured that `CheRRI`'s training data is built from reliable RRI sites with strong evidence.

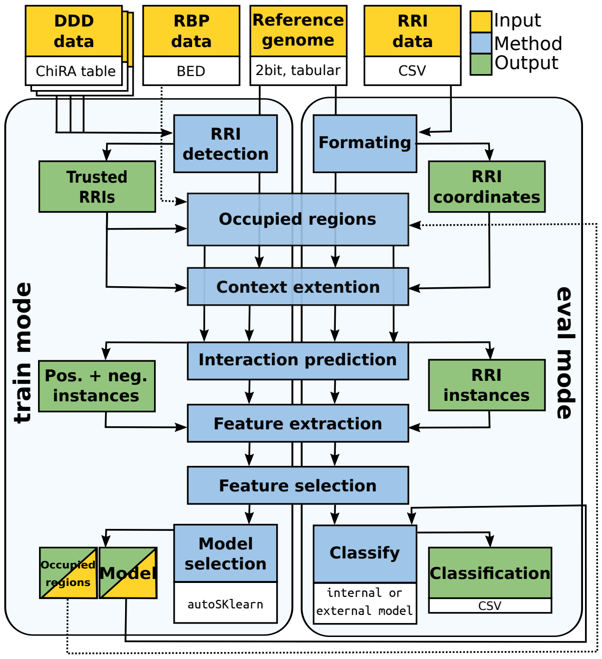

Figure 2. The `CheRRI` workflow. Except for the initial data processing step, both the model selection step in `train` mode (left) and the classification step in `eval` mode (right) use the same core modules. In detail, the `train` mode takes DDD data as input while a tabular file containing RRI sites to be evaluated is provided in the `eval` mode. A reference genome can be automatically downloaded (human and mouse) or needs to be provided for both modes. Optionally, RBP data can be provided as well. After extracting the sequences with context, `CheRRI` uses `IntaRNA` to predict interactions anchored within the sites. Then various features are extracted from the predicted RRIs as well as sequence, context, and accessibility information. These features are then used to either build an organism-specific classification model (in `train` mode) or to evaluate (in `eval` mode) the given RRIs with such a model.

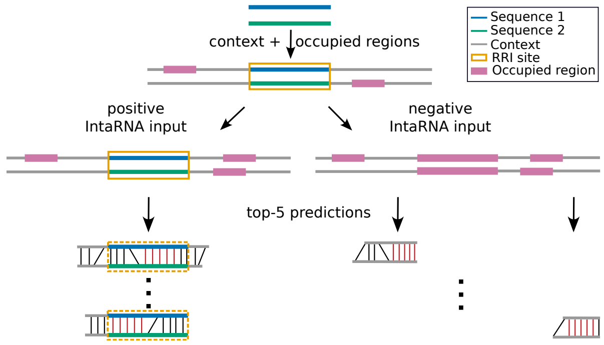

Figure 3. Generating interaction details for reliable RRI sites. Starting with the subsequences defining an RRI site (top blue and green) detected by a direct duplex detection (DDD) method, the sequence is first extended with genomic context (grey). In these extended sequences, all regions known to be occupied e.g. by interaction with other RNAs or proteins are masked as occupied regions (pink boxes). To create interaction details for reliable interactions (positive data), `IntaRNA` predictions are required to show a seed (red base pairs) within the original RRI site (orange box) while avoiding masked regions. For negative interactions, also the RRI site is masked as occupied and "flanking" interactions from the RRI site's genomic context are predicted. In both cases, the top-5 ranked `IntaRNA` predictions are subsequently taken into account to compile the features of an RRI site. The number of suboptimals can be changed by the user.

Occupied regions within the genomic context of RRI sites

`CheRRI` also takes into account whether or not regions in the context of the RRI site of interest are likely occupied, i.e. involved in RNA-RNA or RNA-protein interaction. To this end, regions that are part of sites of the input interactome are considered occupied. The interactions used to build the occupied region library are selected on a lower score cutoff (≥ 0.5), to implement a very conservative definition of "occupied".

Furthermore, the user can provide additional information of other putative binding sites. In detail, `CheRRI` supports the input of RBP binding site information in BED format. The respective regions are also added to the library of occupied regions that are excluded from interaction prediction in the next step.

RRI base pairing details via constraint RRI prediction

Each RRI site will be represented by a set of features that are later assessed by the machine learning model. `CheRRI` takes both the sequence as well as hybridization characteristics of the RRI site and its surrounding genomic context into account. Thus, to investigate the hybridization strength of a putative interaction site, `CheRRI` explores multiple top-ranked `IntaRNA` RRI predictions per site. Investigating a set rather than a single prediction per site reduces the bias of the underlying thermodynamic model and incorporates structural alternatives and flexibility of the interaction. In detail, `IntaRNA` is constrained to identify the five most stable interactions that show a reliable sub-interaction (of 5 consecutive base pairs, called a *seed*) covered by the two subsequences that define the RRI site. Thus, the predictions are anchored by the RRI site but can stretch over its limits into the context. Table S1 gives an overview of the additional `IntaRNA` settings used by `CheRRI`, which can be adapted by the user.

Furthermore, the interactions are also not allowed to cover known occupied regions, i.e. regions that are either other RRI sites or known RBP binding sites (see above). Both constraints are depicted in the lower left of Fig. 3. This is important to give the computational tool the freedom to identify the most-stable base pairing patterns while linking the prediction with the experimental information with limited reliability concerning the site's boundary information.

Creating a sound negative dataset

In order to train and assess the power of our machine learning approach, we need besides the introduced reliable RRI data (our positive dataset) also a set of "non-interacting" data (negative data training). To ensure that our negative data shows similar features as our positive dataset, negative training instances are predicted in close proximity to each positive instance but have to be outside of all other RRI sites. If available, additional interaction information like provided RNA-protein interaction sites are also masked to further enhance the generation of negative data with respective constraints. Negative interactions are then predicted within the not masked context areas as depicted in Figure 3.

The resulting set of negative RRI predictions is neither overlapping with reliable RRI sites nor with known interaction sites, but is likely showing sequence properties and thus interaction features similar to the positive set.

Features, globally and on a neighborhood level

Various features of each `IntaRNA` prediction like interaction strength, accessibility, length, base pairing, etc. are subsequently taken into account. Further sequential features like sequence complexity, GC content, or other nucleotide ratios are generated. More complex sequence features like the sequence complexity of each interaction site as well as normalized features like the energy normalized by the interaction length or the number of GCs are also calculated to represent interactions. The list of features considered by `CheRRI` models is provided in Table S2.

In addition to these global features, graph kernel features are available. Here a graph of all predicted base pairing patterns (multiple per site) is generated, which incorporates both sequence and structure information. From this, (sub)graph features are extracted

via a Weisfeiler-Lehman message passing scheme implemented in EDeN [29] (Explicit Decomposition with Neighborhoods), which allows for the essential link of local structural context (base pairing) with sequence information in RNAs.

Automated selection of a good feature set and classification algorithm including optimized hyperparameters

Depending on the training data and the (graph-)kernel parameters, features might be numerous and ineffectual. To save time in further processing, we directly drop some (very low cutoff) features based on random forest feature importance scores. Feature, model, and hyperparameter optimization is conducted via autoSKLearn [30], which applies efficient Bayesian optimization to find an effective model. autoSKLearn inspects 15 classification algorithms of the python package scikit-learn and is capable to train ensemble models. Per default, all classification estimates are inspected for model selection. However, the list of estimators can be specified by the user.

Model training using different data sources

CheRRI not only enables training based on interactomes from one organism but it also allows to train a combined model for several organisms. To get a combined model, the training mode needs to be run for each individual organism first. In a subsequent training call, using the mixed model mode, the different feature sets will be joined and a mixed model trained. The workflow is detailed in the online documentation linked above.

Applying models on putative RRI sites

In CheRRI's eval mode, see Figure 1, a set of given putative RRI sites can be classified using a provided model. That is their biological relevance is assessed based on the used model. Precomputed models for human and mouse can be downloaded from Zenodo [31]. An output table is computed providing the positions-based identifier of the given RRI sites and the predicted label, i.e. their final assessment of whether or not each site is expected to be biologically functional. It is recommended to also use the library of occupied regions that was generated and used for model training within the eval mode. That way, similar prediction constraints are applied. Furthermore, the same reference genome should be used.

Data used to evaluate CheRRI

The training data was obtained from the DDD method PARIS [11] and further processed using the RNA-RNA interactome analysis tool ChiRA.

ChiRA data preparation. CheRRI's training mode takes as input interaction summary files, which are one of the outputs of the ChiRA workflow. The input data of this study was extracted from the following Galaxy [32] history [33]. Novel data can also be prepared using ChiRA. A respective Galaxy tutorial is available [34].

Build of training instances. Out of the published PARIS data for the human HEK293T cell line and mouse embryonic stem cells dataset [11], four datasets were prepared in total: (1) "Human", (2) "Human + RBP" using RNA-protein interactome data [35], (3) "Mouse", and (4) "Full" by merging the datasets (1+2+3). The RRI sites were filtered by a score of 1 to be considered as "trusted", meaning that only uniquely-mapped reads are mapped to this interaction region. Only RRI sites found in all replicates were kept.

The "Human" and "Mouse" datasets were built using only RRI site information, i.e. only RRIs are considered for the masking of occupied regions (see above). The "Human + RBP" dataset additionally incorporates regions involved in RNA-protein interactions as occupied. For the human HEK cell line, we used the RNA-protein interactome data from [35]. We extracted the crosslink positions of the protein occupancy cDNA libraries from SRA (GEO accession ID GSE38355) in BedGraph format, converted the positions to the hg38 reference genome, and the BedGraph format to BED format. To capture more complete interaction sites, the crosslink positions were extended by 10 nt (i.e., 5 nt up- and downstream). For all datasets, trusted interaction regions were extended left and right by 150 nt genomic context (see Figure 3). Using the occupied region information, a negative dataset was generated for evaluation as described earlier and in supplementary section S1.3. A summary of the dataset sizes can be found in Table 1.

Table 1. Dataset sizes of trusted RRI sites and their respective positive (pos) and negative (neg) RRI instances. The positive and negative instances are the up to 5 IntaRNA predictions per site.

Dataset	trusted RRIs	training data pos	neg
Human	7,780	20,547	32,517
Human + RBP	7,780	14,845	27,744
Mouse	9,594	26,615	36,453

Methods for model validation

CheRRI also provides the option to evaluate the predictive power of a model. To this end, positive and negative instances in form of a validation dataset have to be provided. This can be done if the validation set is completely different from the train and test set, e.g. data from a different species, than the one of the model training. To cross-validate an individual model, a 5-fold cross-validation was used for the results of this publication. This functionality is part of the Auto-sklearn wrapper biofilm [36] that we integrated into CheRRI.

Example calls on how to perform the validation or cross-validation are given in CheRRI's online documentation. The computation of the F1 score, the precision-recall curve (PRC) and the respective tables and plots were performed by scripts provided in CheRRI's GitHub repository and can be used to reproduce the given results.

Results and Discussion

Model training

To train and evaluate CheRRI, the data sets described above were used to create four different models: (1) "human", (2) "human RBP", (3) "mouse", and (4) "full". The latter via merging the datasets (1+2+3). Note, that data sets start with a capital letter and models with a lower letter. The model selection was performed on 5 cores allocating 9GB per thread and giving the selection 18,000s time. All final top-performing models use a histogram-based gradient boosting or a random forest classifier.

Classification beyond thermodynamics

Most RRI prediction algorithms, like IntaRNA or TargetRNA [37] rely on an energy model to compute interactions, and thus the energy, as a measure of interaction stability, becomes the selection criterion for true interactions. To prune false positive predictions from the top-scored interactions, we generate the negative training data for CheRRI on the bases of finding interaction sites that are not known to form an interaction but are still highly scored. This way, CheRRI

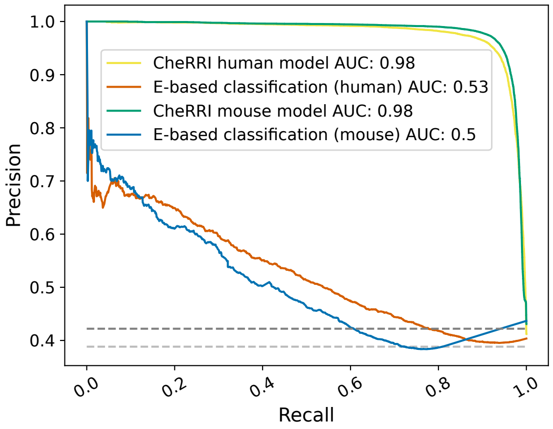

Figure 4. Precision-Recall Plot Comparing CheRRI's models for human and mouse (yellow and green line) against interaction site classification only based on minimal IntaRNA energy scores (E-based classification, orange (human) and blue line (mouse)). The dark grey line shows the baseline, e.g. how many predictions are expected to occur by chance, for the human data-based models and the lighter grey line for the mouse data-based models.

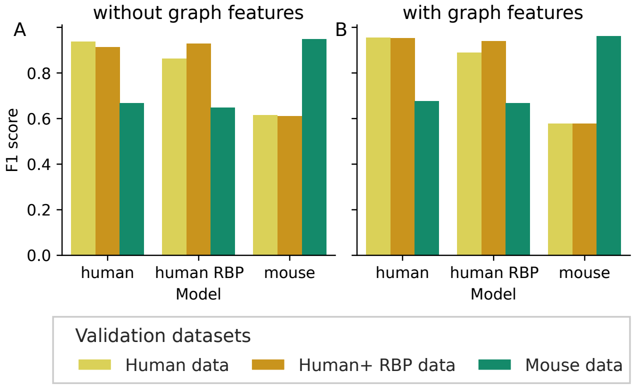

Figure 5. Evaluation of models The model performance is measured by the F1 score, using different validation datasets. The figure compares the "Human", "Human + RBP", and "Mouse" dataset. On the left side (A) without graph-kernel models and (B) on the right side including graph-kernel features. The model validation was performed using 5-fold cross-validation. All training data not used for a particular model training were used for validation, e.g. human model validated on mouse data.

is able to learn features that identify functional RRIs beside stability and avoid a strong bias towards interaction energy.

We assessed this hypothesis via a comparison of the predictive power of our models against a simple classification only based on minimal interaction energy per putative site reported by IntaRNA (E-based classification). Figure 4 summarizes our findings for the Human and Mouse datasets in terms of precision-recall curves (PRCs). There is a clear distinction between the energy-based classification vs. CheRRI's models with additional features. As we can see, energy alone is not sufficient to distinguish functional from false positive RRIs. The supplementary data shows the PRCs for models with graph-kernel features in Figure S3 and the AUC results in Table S7 and S9. The energy-based classification perform with 0.53 and 0.5 a bit better than random guessing, see grey baselines. The CheRRI models both human and mouse have a very high detection rate (both 0.98 AUC).

Given the feature importance analysis detailed in the supplementary material, GC content, and sequence complexity are among the most important features that distinguish the CheRRI model from the energy-only model, besides GC- and length-normalized features.

Model comparison

Model performance (in terms of F1 score) was evaluated by 5-fold cross-validation or on datasets not used for training (see Table S6 and S8). The incorporation of graph-kernel features encoding the RRI base-pairing improved the F1 score of the cumulative model "full" from 0.94 (no graph-kernel features) to 0.95 (with graph-kernel features). The best performance was observed for the "human" model when applied to "Human + RBP" data incorporating RNA-protein interaction sites (0.95/0.91 F1 score with/without graph-kernel, resp.). Even evaluating "Mouse" interaction sites with the "human" model shows reasonable results (0.68/ 0.67 F1 score with/without graph-kernel, resp.). Using a "mouse" model for "Human" RRI evaluation still showed moderate F1 scores (0.59/0.61). The F1 comparisons can be seen in Figure 5.

The "mouse" model can distinguish known interactions from the ones which are not present better than the "human" model (F1 score for human/mouse with and without graph-kernel: 0.95/0.96 and 0.94/0.95). The number of RRIs used for building training instances is higher for "mouse" (9,594) compared to "human" (7,780), see Table 1. However, applied to RRI sites of a different organism the "human" model performs better.

When investigating the difference between human and mouse models in terms of feature importance (see supplementary material), we see strong importance differences for energy and GC-content features, while length-normalized variants show much lower deviations. This could result from a bias in one of the data sets towards specific classes of interactions or sequences and shows the known data-sensitivity of machine-learning based models that can easily lead to overfitting. Thus we conclude, that revised data sets might reduce this limited portability of the tested organism-specific models. This hypothesis is also supported by the results of the "full" model that is trained on a combination of human and mouse data. This model shows excellent performance for all data sets in terms of F1 scores, which is shown in Tab. S5 and S7 of the supplementary material.

Ad hoc usability via Galaxy

CheRRI is easily available via pip and conda package managers, via the bioconda channel [38], for command line usage and integration into data analysis workflows. To provide a graphical interface for an interactive user-friendly experience, CheRRI can also be found on Galaxy [32]. All results presented within the manuscript are created via the Galaxy interface, which enables full reproducibility. All models trained on Galaxy are accessible via a Galaxy history [39]. CheRRI is publicly available on GitHub and can be used under a GPL-3.0 license [40].

Conclusion

We introduce CheRRI, the first post-processing pipeline to assess the biological relevance of putative RRI sites. Typically, RRI sites are assessed based on their minimum free energy, which is only a poor classifier. In contrast, CheRRI learns and applies highly accurate classification models that incorporate detailed features of RRIs and their context derived from reliable experimental interactome data. The latter should be filtered via RNA-RNA interactome evaluation pipelines like ChiRA or RNANUE [41] prior to using CheRRI to focus on highly reliable data.

CheRRI is easy to install (e.g., via conda) and flexible to use due to its built-in feature and model optimization. The Galaxy integration directly allows its application in reproducible workflows. Currently, CheRRI provides pre-built models for human and mouse based on PARIS data (filtered using ChiRA). All models and feature sets are

publicly available along with `CheRRI` and can be used to evaluate any given set of RRI sites.

Our analyses show that our models are still organism and data specific. This might result from an unknown bias within the data, the restriction to a specific experimental protocol or the still limited training data size. The flexibility to extend the data and to curate and use own project-specific data sets is a central strength of `CheRRI`. Therefore extended and revised data sets will continuously improve its reliability.

Availability of source code and requirements

- Project name: CheRRI
- Project home page: https://github.com/BackofenLab/Cherri
- Operating system(s): CL-tool, Galaxy
- Programming language: Python
- License: GPL-3.0 license
- BioToolsID: biotools:cherri
- RRID: SCR_025175

An archival copy of the code is available via Software Heritage [42]

Data Availability

The generated models as well as data supporting the results of this article are available in the Zenodo repository [31].

Declarations

List of abbreviations

Below all abbreviations used for this publication are listed in alphabetical order. Lists the following:

- AUC: area under the curve
- DDD: direct duplex method
- miRNA: micro RNA
- ML: machine learning
- ncRNAs: non-coding RNAs
- nt: nucleotides
- PRC: precision-recall curve
- RBP: RNA-binding protein
- RRI: RNA-RNA interaction
- sRNA: small regulatory RNA

Ethical Approval (optional)

Not applicable

Consent for publication

Not applicable

Competing Interests

The author(s) declare that they have no competing interests.

Funding

Bundesministerium für Bildung und Forschung, RNAProNet, 031L0164B, R Backofen; Deutsche Forschungsgemeinschaft, Collaborative Research Centers, SFB-1425/1, R Backofen; Deutsche Forschungsgemeinschaft, Excellence Strategy, EXC-2189 (CIBSS), R Backofen; Deutsche Forschungsgemeinschaft, Sachbeihilfe, BA 2168/14-1, R Backofen;

Author's Contributions

T.M. designed the study, collected and curated the data, implemented the tool, trained and evaluated the models, and wrote the manuscript. R.B., F.E., and M.R. helped in designing the study. S.M. provided the biofilm pipeline. T.M. and P.V. implemented the `Galaxy` wrapper. All authors contributed to the writing and revising of the manuscript.

Acknowledgements

We would like to thank Sebastian Holler for the preliminary investigations and the reviewers for their valuable comments on the manuscript. The Freiburg Galaxy Team and the European Galaxy Server are supported by the Ministry of Science, Research and the Arts Baden-Württemberg (MWK) within the framework of LIBIS/de.NBI Freiburg This work was supported by the BMBF-funded de.NBI Cloud within the German Network for Bioinformatics Infrastructure (de.NBI) (031A532B, 031A533A, 031A533B, 031A534A, 031A535A, 031A537A, 031A537B, 031A537C, 031A537D, 031A538A).

References

1. Guil S, Esteller M. RNA–RNA interactions in gene regulation: the coding and noncoding players. Trends in biochemical sciences 2015;40(5):248–256.
2. Bunch H. Gene regulation of mammalian long non-coding RNA. Molecular Genetics and Genomics 2018;293(1):1–15.
3. Pu M, Chen J, Tao Z, Miao L, Qi X, Wang Y, et al. Regulatory network of miRNA on its target: coordination between transcriptional and post-transcriptional regulation of gene expression. Cellular and Molecular Life Sciences 2019;76(3):441–451.
4. Hör J, Gorski SA, Vogel J. Bacterial RNA biology on a genome scale. Molecular cell 2018;70(5):785–799.
5. Desgranges E, Caldelari I, Marzi S, Lalaouna D. Navigation through the twists and turns of RNA sequencing technologies: Application to bacterial regulatory RNAs. Biochimica et Biophysica Acta (BBA)-Gene Regulatory Mechanisms 2020;1863(3):194506.
6. Panni S, Lovering RC, Porras P, Orchard S. Non-coding RNA regulatory networks. Biochimica et Biophysica Acta (BBA)-Gene Regulatory Mechanisms 2020;1863(6):194417.
7. Gong J, Shao D, Xu K, Lu Z, Lu ZJ, Yang YT, et al. RISE: a database of RNA interactome from sequencing experiments. Nucleic acids research 2018;46(D1):D194–D201.
8. Lin Y, Liu T, Cui T, Wang Z, Zhang Y, Tan P, et al. RNAInter in 2020: RNA interactome repository with increased coverage and annotation. Nucleic acids research 2020;48(D1):D189–D197.
9. Weidmann CA, Mustoe AM, Weeks KM. Direct duplex detection: an emerging tool in the RNA structure analysis toolbox. Trends in biochemical sciences 2016;41(9):734–736.
10. Lin C, Miles WO. Beyond CLIP: advances and opportunities to measure RBP–RNA and RNA–RNA interactions. Nucleic acids research 2019;47(11):5490–5501.
11. Lu Z, Zhang QC, Lee B, Flynn RA, Smith MA, Robinson JT, et al. RNA duplex map in living cells reveals higher-order transcriptome structure. Cell 2016;165(5):1267–1279.
12. Sharma E, Sterne-Weiler T, O'Hanlon D, Blencowe BJ. Global mapping of human RNA-RNA interactions. Molecular cell 2016;62(4):618–626.
13. Aw JGA, Shen Y, Wilm A, Sun M, Lim XN, Boon KL, et al. In vivo mapping of eukaryotic RNA interactomes reveals principles

of higher-order organization and regulation. Molecular cell 2016;62(4):603–617.
14. Schönberger B, Schaal C, Schäfer R, Voß B. RNA interactomics: recent advances and remaining challenges. F1000Research 2018;7.
15. Fukunaga T, Hamada M. RIblast: An ultrafast RNA-RNA interaction prediction system based on a seed-and-extension approach. Bioinformatics (Oxford, England) 2017;33:2666–2674.
16. Alkan F, Wenzel A, Palasca O, Kerpedjiev P, Rudebeck A, Stadler P, et al. RIsearch2: Suffix array-based large-scale prediction of RNA-RNA interactions and siRNA off-targets. Nucleic acids research 2017;45:e60.
17. Mann M, Wright PR, Backofen R. IntaRNA 2.0: enhanced and customizable prediction of RNA-RNA interactions 2017;45(W1):W435–W439.
18. Lai D, Meyer IM. A comprehensive comparison of general RNA-RNA interaction prediction methods. Nucleic acids research 2016;44(7):e61–e61.
19. Umu SU, Gardner PP. A comprehensive benchmark of RNA–RNA interaction prediction tools for all domains of life. Bioinformatics 2017;33(7):988–996.
20. Raden M, Müller T, Mautner S, Gelhausen R, Backofen R. The impact of various seed, accessibility and interaction constraints on sRNA target prediction-a systematic assessment. BMC bioinformatics 2020;21:1–11.
21. Fang Y, Pan X, Shen HB. Recent Deep Learning Methodology Development for RNA–RNA Interaction Prediction. Symmetry 2022;14(7):1302.
22. Lu ZJ, Mathews DH. Efficient siRNA selection using hybridization thermodynamics. Nucleic acids research 2008;36(2):640–647.
23. Liu C, Mallick B, Long D, Rennie WA, Wolenc A, Carmack CS, et al. CLIP-based prediction of mammalian microRNA binding sites. Nucleic acids research 2013;41(14):e138–e138.
24. Kim SK, Nam JW, Rhee JK, Lee WJ, Zhang BT. miTarget: microRNA target gene prediction using a support vector machine. BMC bioinformatics 2006;7(1):1–12.
25. Tjaden B. TargetRNA3: predicting prokaryotic RNA regulatory targets with machine learning. Genome Biology 2023;24:276.
26. Reynolds A, Leake D, Boese Q, Scaringe S, Marshall WS, Khvorova A. Rational siRNA design for RNA interference. Nature biotechnology 2004;22(3):326–330.
27. Møller T, Franch T, Højrup P, Keene DR, Bächinger HP, Brennan RG, et al. Hfq: a bacterial Sm-like protein that mediates RNA-RNA interaction. Molecular cell 2002;9(1):23–30.
28. Videm P, Kumar A, Zharkov O, Grüning BA, Backofen R. ChiRA: an integrated framework for chimeric read analysis from RNA-RNA interactome and RNA structurome data. GigaScience 2021;10(2):giaa158.
29. Navarin N, Costa F. An efficient graph kernel method for non-coding RNA functional prediction. Bioinformatics 2017 05;33(17):2642–2650.
30. Feurer M, Klein A, Eggensperger J Katharina Springenberg, Blum M, Hutter F. Efficient and Robust Automated Machine Learning. In: Advances in Neural Information Processing Systems 28 (2015); 2015. p. 2962–2970.
31. Müller T, Mautner S, Videm P, Eggenhofer F, Raden M, Backofen R, Cherri - Accurate detection of functional RNA-RNA interactions sites (cherri v0.8). Zenodo; 2024. https://doi.org/10.5281/zenodo.10555733.
32. Afgan E, Baker D, Batut B, Van Den Beek M, Bouvier D, Čech M, et al. The Galaxy platform for accessible, reproducible and collaborative biomedical analyses: 2018 update. Nucleic acids research 2018;46(W1):W537–W544.
33. Videm P, Chira - PARIS analysis; 2020. https://rna.usegalaxy.eu/u/videmp/h/paris-analysis, history. GalaxyEU.
34. Videm P, Chira - RNA-RNA interactome data analysis; 2020. https://training.galaxyproject.org/training-material/topics/transcriptomics/tutorials/rna-interactome/tutorial.html, training materials. Galaxy Training Network.
35. Baltz AG, Munschauer M, Schwanhäusser B, Vasile A, Murakawa Y, Schueler M, et al. The mRNA-bound proteome and its global occupancy profile on protein-coding transcripts. Molecular cell 2012;46(5):674–690.
36. Mautner S. Biofilm. GitHub repository 2021;https://github.com/smautner/biofilm.
37. Kery MB, Feldman M, Livny J, Tjaden B. TargetRNA2: identifying targets of small regulatory RNAs in bacteria. Nucleic acids research 2014;42(W1):W124–W129.
38. Grüning B, et al. Bioconda: sustainable and comprehensive software distribution for the life sciences. Nature methods 2018;15(7):475–476.
39. Müller T, Mautner S, Videm P, Eggenhofer F, Raden M, Backofen R, CheRRI - Trained models on Galaxy; 2024. https://usegalaxy.eu/u/videmp/h/cherri-model-training, history. GalaxyEU.
40. Müller T, Mautner S, Videm P, Eggenhofer F, Raden M, Backofen R. Cherri. GitHub repository 2020;https://github.com/BackofenLab/Cherri.
41. Schäfer RA, Voß B. RNAnue: efficient data analysis for RNA–RNA interactomics. Nucleic acids research 2021;49(10):5493–5501.
42. Müller T, Mautner S, Videm P, Eggenhofer F, Raden M, Backofen R, CheRRI - Accurate classification of the biological relevance of putative RNA-RNA interaction sites (Version 0.8); 2024. https://archive.softwareheritage.org/swh:1:snp:ebac091117f9c46fb5f0fedd3ef23ec2905ced6c;origin=https://github.com/BackofenLab/Cherri, available online. [Computer software]. Software Heritage.

Fig. 3 supplement

Click here to access/download;Figure;benchmark-workflow.png

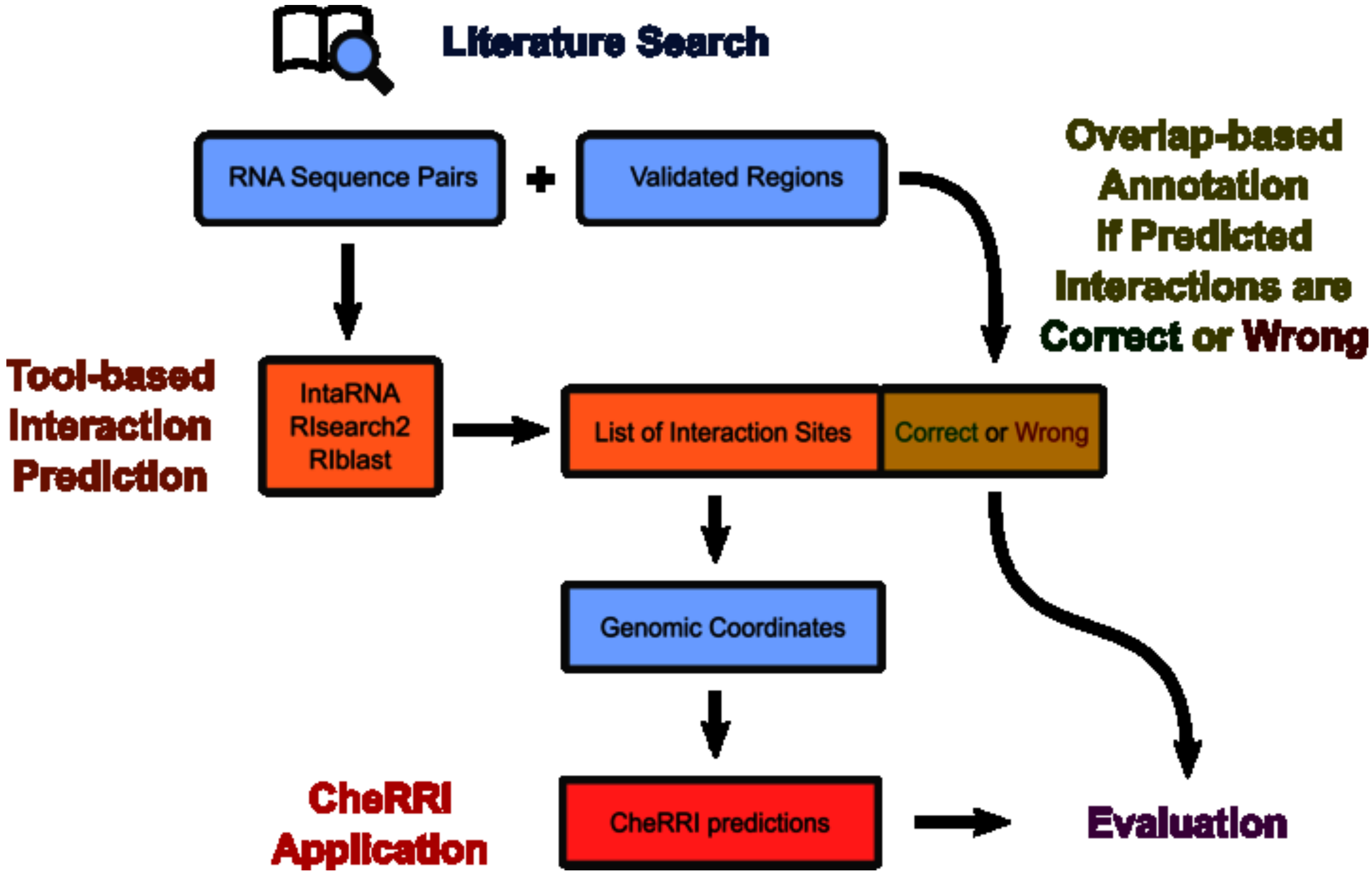

Fig. 2 main paper

Click here to access/download;Figure

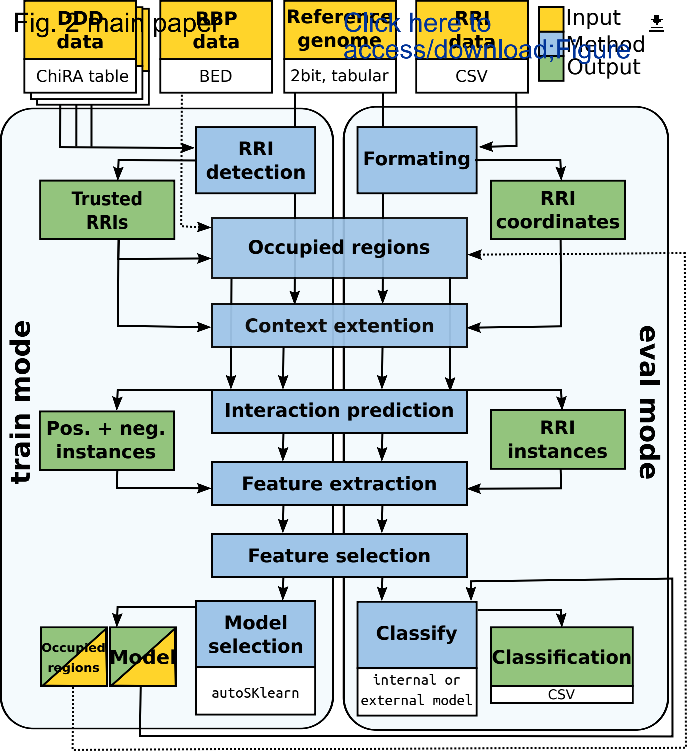

Fig. 3 main paper

Click here to access/download;Figure;data_generation.pdf

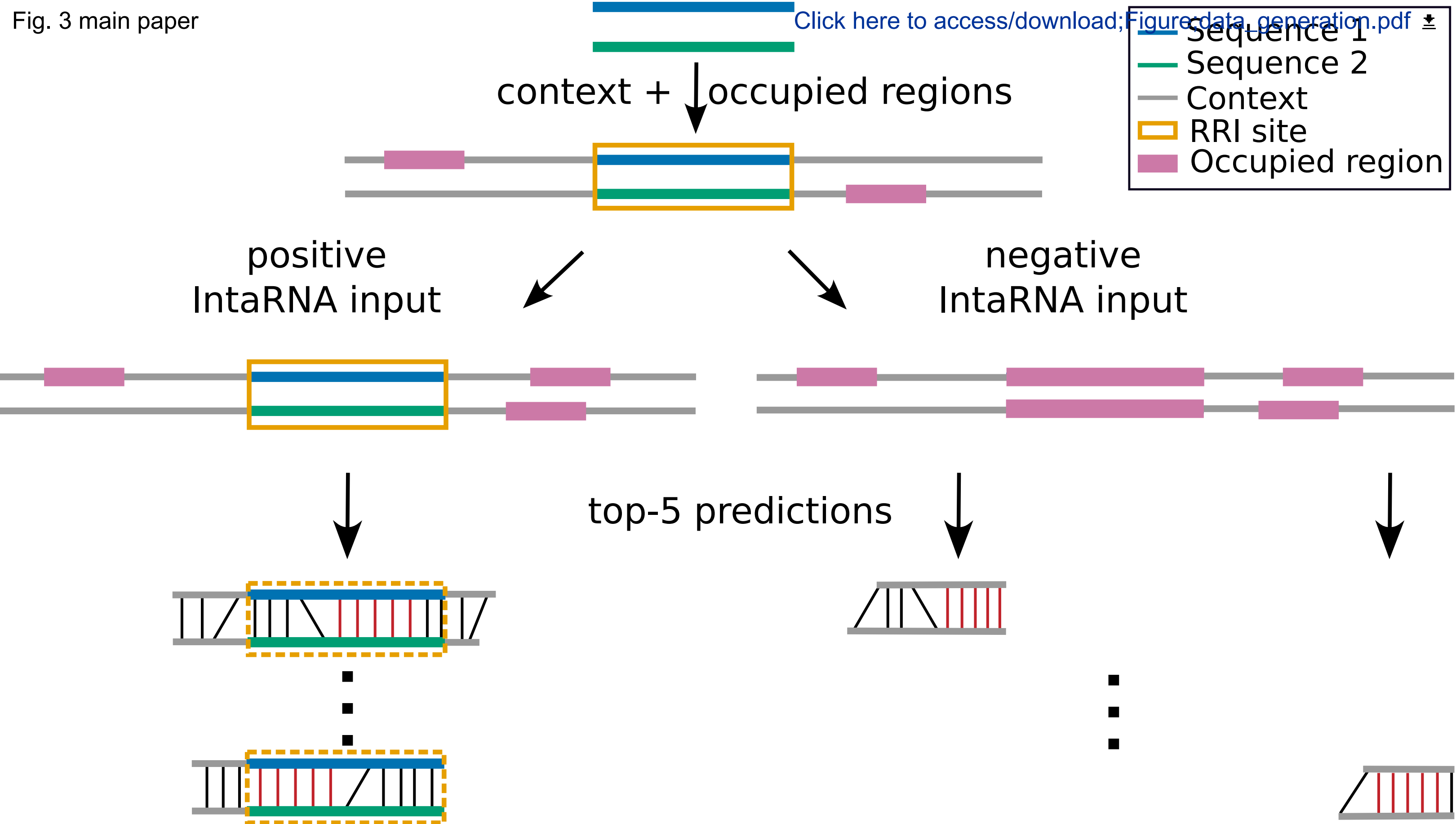

Fig. 4 main paper

Click here to access/download;Figure;prc_plot.pdf

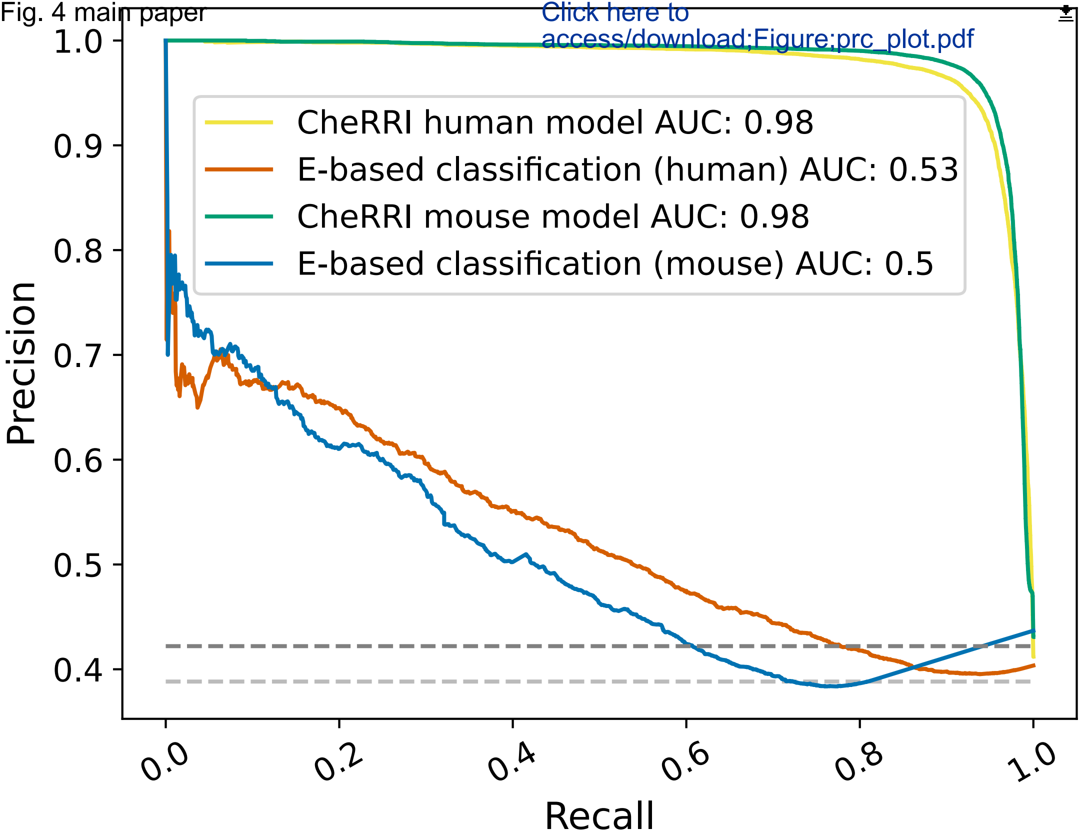

Click here to access/download;Figure;CheRRI_main_fig_2.pdf

Fig. 1 main paper

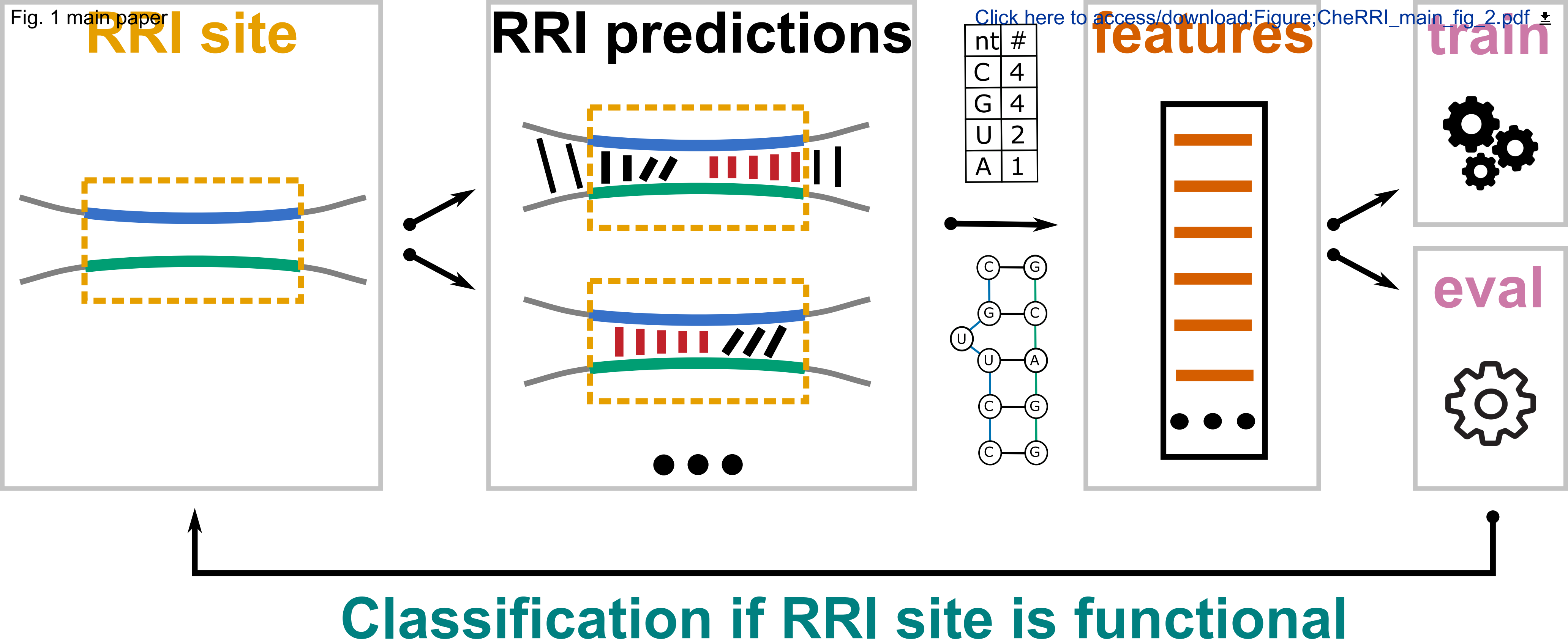

Fig. 5 main paper

Click here to access/download;Figure;F1_cross_model

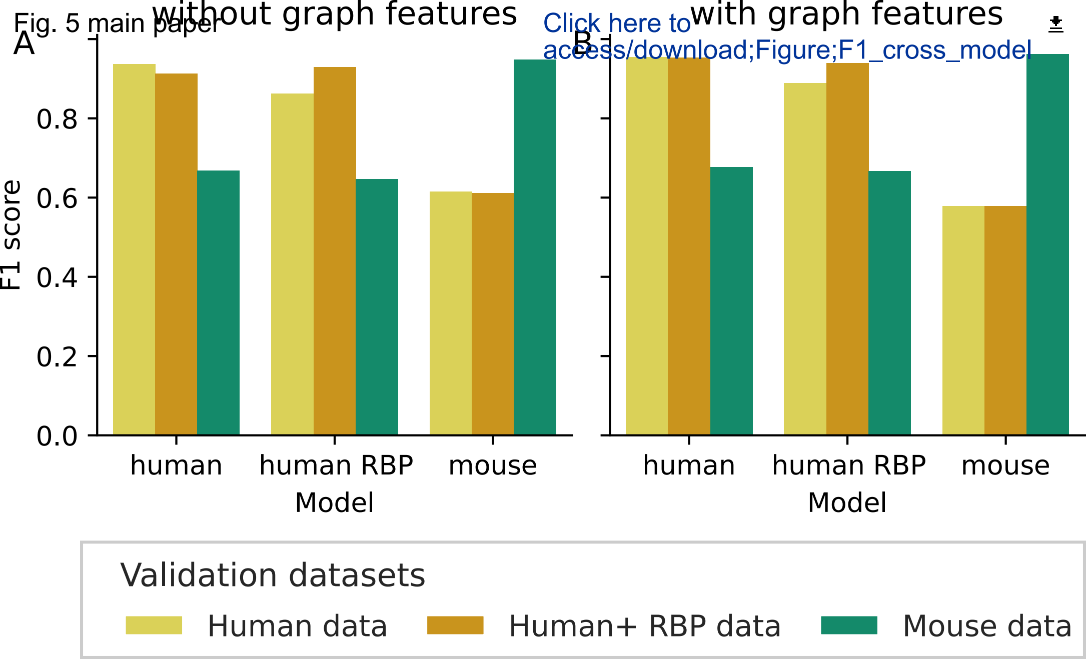

Fig. 2 supplement

Click here to access/download;Figure;prc_plot_supp.pd

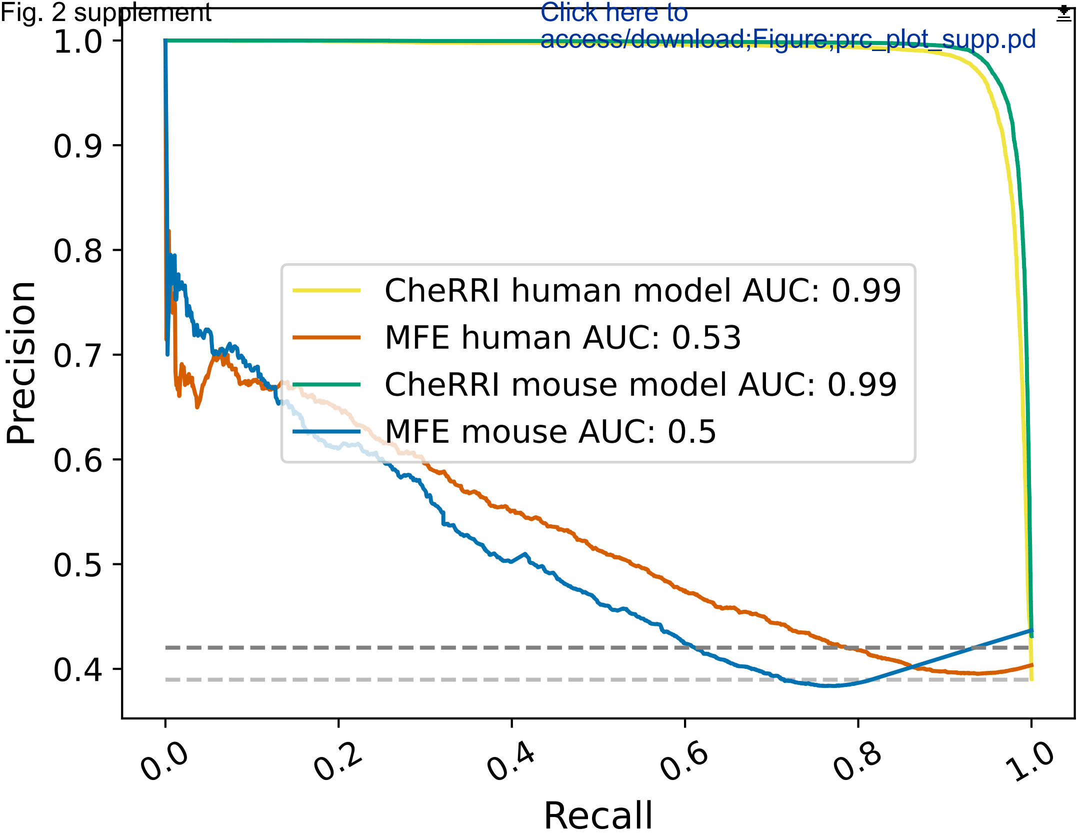

Fig. 1 supplement

Click here to access/download;Figure;feature_importance_barplot_mouse_human.pdf

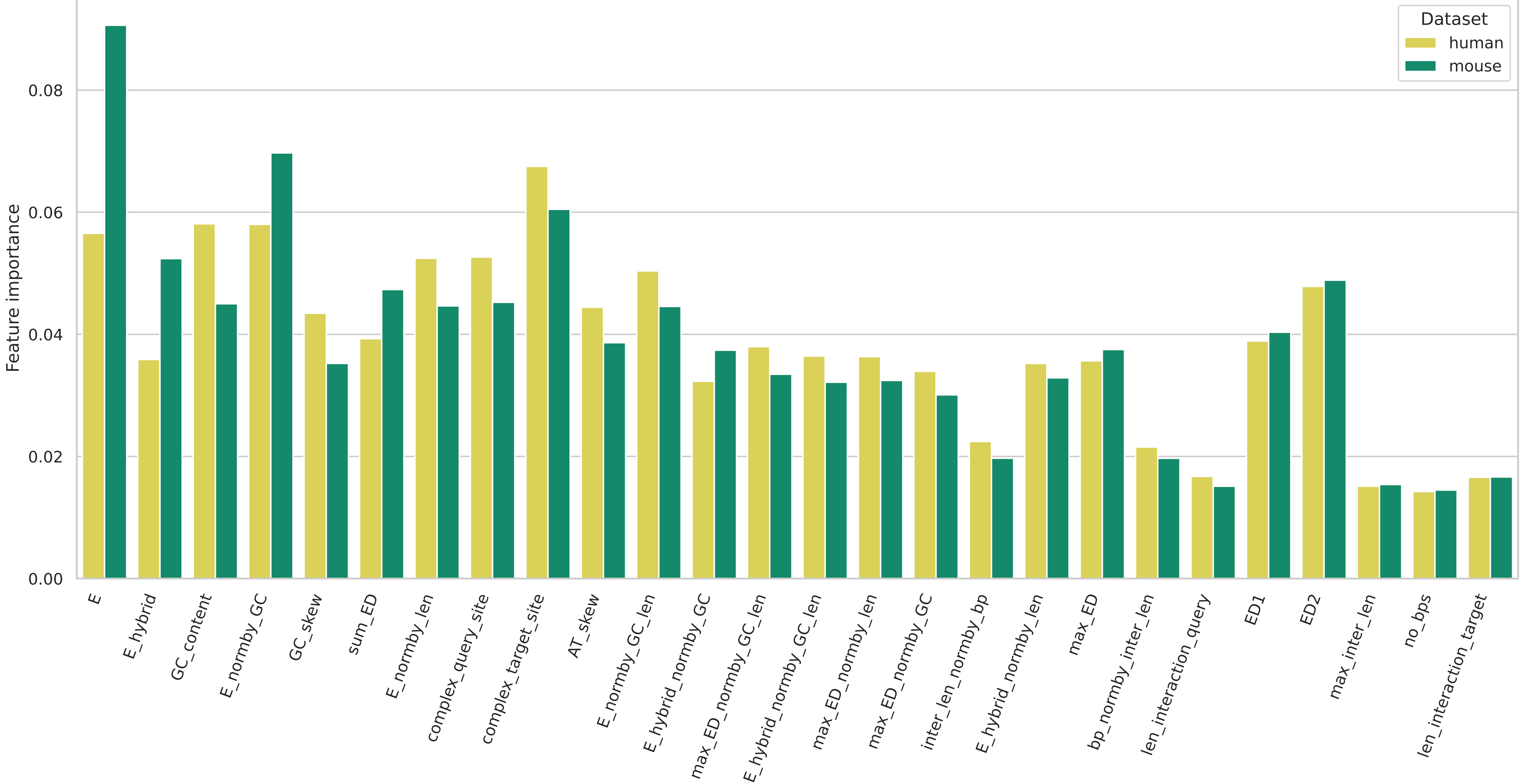

Manuscript

Click here to access/download
Supplementary Material
main.tex

LaTeX - support file

Click here to access/download
Supplementary Material
oup-contemporary.cls

LaTeX - support file

Click here to access/download
Supplementary Material
vancouver-authoryear.bst

latex Bib of the supplement

Click here to access/download
Supplementary Material
supplement.bib

Supplement tex file

Click here to access/download
Supplementary Material
supplement.tex

latex Bib of the main paper

Click here to access/download
Supplementary Material
paper-refs.bib

Click here to access/download
Supplementary Material
Cherri_GigaScience_final_submission_supplement.pdf

make file for the figs folder which contains all figs of the main paper

Click here to access/download
Supplementary Material
Makefile
