# Supplementary material for: CheRRI—Accurate classification of the biological relevance of putative RNA–RNA interaction sites
Source: Gigascience. 2024 Jun 5;13:giae022. doi: 10.1093/gigascience/giae022 (PMC11152173; doi:10.1093/gigascience/giae022)
Supplement: giae022_Response_to_Reviewer_Comments_Original_Submission [file giae022_response_to_reviewer_comments_original_submission.pdf]

Author's Response To Reviewer Comments

Dear editor Hans Zauner,

many thanks for giving us the opportunity to revise our technical note in the light of the reviewers' comments.

We have addressed all concerns and suggestions provided by the reviewers. You can find the point-by-point answers to their comments below. In detail, we conduct a thorough feature importance analysis with respective discussions within the manuscript. Furthermore, we have curated and applied an independent benchmark data set and have textually revised central parts of the manuscript to incorporate the reviewers' comments.

We are looking forward to receiving the reviewers' assessment of our changes and your feedback on our technical note.

Sincerely yours,
on behalf of all authors,
Teresa Müller

Reviewer Comments and Replies
==============================

In the following, we hope to answer any outstanding questions. Changes in the main manuscript and supplemental materials are marked in red.

Reviewer 1
=============

COMMENT:

In this study, the authors have developed CheRRI, a machine learning-based software to predict RNA-RNA interactions using large-scale interactome data as the training input. Previous methods for predicting RNA-RNA interactions have relied on the RNA energy models, which had moderate but not high accuracy. The approach of improving prediction accuracy by integrating machine learning with large-scale experimental data and RNA energy models should be an effective strategy, and thus the research direction is highly important. However, this study did not involve sufficient experiments or analysis to demonstrate the usefulness of the software.

ANSWER:

We thank the reviewer for his/her thorough review of our work.
Unfortunately, given the reviewer's summary, we noted that the description of our method seems to be still not fully to the point, since the reviewer wrongly assumes CheRRI is tailored to predict RNA-RNA interactions. That is not the case!
CheRRI was built as a filter to classify given RNA-RNA interactions on a genomic scale that could stem from any source, e.g. from wet-lab protocols or from in-silico predictions. While internal feature generation also involves localized interaction prediction, CheRRI itself does not predict new interaction sites but rather classifies given sites as reliable or not.
We have thus revised our introduction and the graphical abstract in Figure 1 to clarify this objective.
Furthermore, we have extended our analyses of CheRRI in the light of the reviewer's suggestions, as detailed below, and thank the reviewer for his/her ideas and support.

COMMENT:

1. In the fourth paragraph of the Introduction, the authors stated "genome-wide applications are currently out of reach" and "filtering the predictions for functional candidates, specifically on a transcriptome-wide scale, remains an unsolved challenge". I understood that the authors developed CheRRI to solve this problem. CheRRI requires interaction prediction results by IntaRNA as the input, and thus the prediction by IntaRNA is first needed. However, for the large-scale data analysis, the filtering should be performed before the prediction by IntaRNA, which is computationally time-consuming. In other words, this method has not achieved the application of IntaRNA to the transcriptome level. In addition, because there are now methods for predicting RNA-RNA interactions at the transcriptome level, such as RIsearch2 and RIblast, the discussion in the introduction is not sufficiently based on previous research.

ANSWER:

Thanks for pointing this out. We have thoroughly revised and extended the paragraph and bibliography, respectively.
As discussed above, CheRRI is designed to be applied as a post-prediction or post-experiment filter to prune false positive RRI candidates. Thus, its effect and application is orthogonal to the efforts spent to improve in-silice tools and wet-lab protocols. We totally agree that IntaRNA cannot be applied as it is in a high-throughput full genome wide screen to identify all possible interaction partners. But this is neither done nor intended within the CheRRI pipeline. In contrast, CheRRI is intended to be applied to an already pruned set of candidates (eg. based on prediction scores or other measurements). Furthermore, we apply the highly accurate (but computationally costly) IntaRNA tool only in a very specific and localized manner, i.e. anchoring predictions at the restricted set of the given putative interaction sites of interest that have to be classified.
Since this point was not well receivable, we have added and extended respective paragraphs within the introduction.

COMMENT:

2. The authors showed that the AUPR of CheRRI is sufficiently higher than the AUPR of MFE in the Fig. 4. However, the authors did not provide the sufficient analysis of which features contributed to the higher accuracy of the predictions. The authors should analyze features that are effective for classification, e.g. through the feature importance analysis and SHAP analysis. In particular, it would be interesting to analyze in what ways humans and mice differ (or have in common). Depending on the features obtained from this analysis, methods such as pre-filtering before the prediction by IntaRNA or incorporating the features to make IntaRNA more accurate should be suggested.

ANSWER:

Again, thanks for the valuable suggestions. We have added a detailed feature importance analysis to the supplementary material and have extended the main text respectively.
Eventually, the reviewer raises two independent questions, while both can be answered with the graph provided in the supplementary material (Fig. S1). It shows the feature importance of all handcrafted features that are part of all our models, sorted by decreasing difference between human and mouse models.
Therein, we can see that within both models GC-content, complexity, and length normalization (and combinations with these) are most important besides energy (E, E_hybrid) and should thus be responsible for the improved performance compared to an energy-only model.
Furthermore, we can see that features that are not length-normalized show the highest differences. This suggests that the length features of IntaRNA interactions predicted in human and mouse data differ, which can be a result of a bias of one or the other data set to specific classes of interaction sites.
We have added a respective discussion of both questions to the main manuscript.

COMMENT:

3. Because this study used the interaction results from a specific experiment as training data, it may be learning the experimental bias rather than the intrinsic nature of the RNA-RNA interaction. The authors should apply CheRRI to existing RNA-RNA interaction benchmark tests and show that the results obtained with the present method are not experimental bias.

ANSWER:

We totally agree, all machine-learning methods are prone to overfitting, i.e. learning a bias present in the training material and not the generic ideal targeted. The same holds for CheRRI, which can be already observed in the limited "portability" of the models to other organisms.
On the other hand, this is one of the central objectives and strengths of CheRRI, since it is tailored not to be only used with the provided pre-defined models but designed to create new models for specific needs and data. That is, we strongly encourage the users to extend our data sets or compile new ones, since upcoming experimental data will definitely boost reliability of CheRRI-based models.
A respective discussion was added to the main manuscript.
Furthermore, we extended our efforts to provide an unbiased benchmark of CheRRI. Unfortunately, we are not sure which "existing RNA-RNA interaction benchmark tests" the reviewer refers to.
To benchmark CheRRI, we need experimentally verified (genomic) localization information of interactions in human or mouse. To the best of our knowledge, we are now aware of nor could find any such data set. RNA interaction databases only provide sequence pairs suitable to study interaction networks but missing the detailed localization information of interaction sites needed for our purpose.
To remedy the problem, we implemented a benchmark workflow that is based on a small set of human RNA-RNA interactions used within the benchmark study by Umu and Gardner (2017). Since this data only provides interaction localizations for target molecules, we generate a set of putative interaction sites via three state-of-the-art prediction tools (RIblast, RIsearch2, IntaRNA). Given the target localization information from literature, we can thus compare CheRRIs classification with the experimental data. The benchmark is provided within the supplementary material and shows that CheRRI provides good and conservative classifications. Due to various limitations of the benchmark, also discussed in the supplement, we refrained from adding it to the main manuscript.
While such a "gold standard benchmark data set" would be most beneficial and appreciated by the whole RRI bioinformatics community, we see its collection well beyond the scope and format of this technical note.

=============
2. Reviewer 2
=============

COMMENT:

In "CheRRI - Accurate classification of the biological relevance of putative RNA-RNA interactions sites," Müller et al. report a new machine learning model to classify putative RNA-RNA interactions as true positive or false positive. The method is able to post-process predictions made by thermodynamics to improve the prediction accuracy for RNA-RNA interactions.

ANSWER:

Thanks to the reviewer for the comprehensive summary. We would like to add that CheRRIs field of application is not limited to in-silico methods based on thermodynamic models but allows to filter any

genomic RNA-RNA interaction site information, e.g. also resulting from wet-lab protocols or other pipelines.

COMMENT:

Minor Revisions:
I like the premise of the work. RNA-RNA interaction prediction identifies many false positives, and a machine learning approach to remove false positives is welcome. I have some concerns about presentation that should be revised before publication.

The precision-recall curves in Figure 4 compare the free energy of MFE structure prediction to CheRRI for human and mouse. The MFE prediction is not the best baseline; the most accurate methods (including IntaRNA) consider the RRI free energy change and the accessibility to binding. Additionally, the negative examples are generated from examples that are "highly scored," which I understood to mean the IntaRNA score. In this way, the negative set was designed to be a challenge for free energy methods. To address these concerns, Figure 4 should be generated using the full IntaRNA model and not just the MFE free energy as the baseline performance. The text should also clarify that CheRRI is designed to post process the thermodynamic prediction, therefore Figure 4 is not a comparison of two methods, but a demonstration of how CheRRI augments the thermodynamics. The current text suggests that it is a comparison of distinct methods.

ANSWER:

Many thanks to pointing this out. We think it is more a problem of writing, since eventually the “energy-based classification” (as it is renamed now) is exactly as the reviewer suggests: the classification of interaction sites solely based on the energy of the best interaction reported by IntaRNA (which is in the end the minimal free energy per interaction site and already includes accessibility terms etc.).
We totally agree that our caption and text was misleading w.r.t. main topic of the section. We have thus renamed the section to “Classification beyond thermodynamics” and revised the text.

COMMENT:

My second concern is with the loss of performance when transferring the model between organisms (mouse to human and human to mouse). This suggests that the model is not completely transferable. I would have hypothesized that the features would be universal, at least within mammals. There is some cursory discussion that the transferability is not high, but the manuscript should address this as a concern.

ANSWER:

Yes, the limited “portability” between organisms was unexpected but is most likely a result of some bias in one or both data sets in combination with overfitting. The effect is strongly reduced when combining human and mouse data for training. Respective data is now more prominently shown and discussed within the manuscript.

COMMENT:

This work has some similarities to earlier work that identified features for siRNA or miRNA target determination and then used those features to predict target sites [Nat Biotech. 2004. 22: 326; NAR. 2008. 36: 640; NAR. 2013. 41: e138]. This manuscript might cite this prior work and explain that CheRRI is a more general approach for target site prediction.

ANSWER:

We agree and have extended our bibliography and discussion of related computational methods.

COMMENT:

In the supplement, S2 provides a list of features that are used. The list is not completely obvious, and some additional description would be helpful. For example, I assume the Shannon entropy is a mean entropy for nucleotides calculated from base pairing probabilities. However, this is not completely clear, and the text does not state how large a folding region is used for estimating the pair probabilities. Additionally, I assume the terms with GC content use the GC content just for the binding region, but again that is not completely clear.

ANSWER:

Thanks to the reviewer for this suggestion. We have extended the information both within the table and within the description within the supplementary material.
