# Supplementary material for: CheRRI—Accurate classification of the biological relevance of putative RNA–RNA interaction sites
Source: Gigascience. 2024 Jun 5;13:giae022. doi: 10.1093/gigascience/giae022 (PMC11152173; doi:10.1093/gigascience/giae022)
Supplement: giae022_Response_to_Reviewer_Comments_Revision_1 [file giae022_response_to_reviewer_comments_revision_1.pdf]

Author's Response To Reviewer Comments

Dear Hans,

Thank you again for accepting and processing our publication. We addressed all of your formatting and editorial points.

Best wishes,
Teresa
