# Supplementary material for: CheRRI—Accurate classification of the biological relevance of putative RNA–RNA interaction sites
Source: Gigascience. 2024 Jun 5;13:giae022. doi: 10.1093/gigascience/giae022 (PMC11152173; doi:10.1093/gigascience/giae022)
Supplement: giae022_Reviewer_1_Report_Original_Submission — Tsukasa Fukunaga -- 11/15/2023 [file giae022_reviewer_1_report_original_submission.pdf]

Reviewer Report

Title: CheRRI - Accurate classification of the biological relevance of putative RNA-RNA interaction sites.

Version: Original Submission **Date:** 11/15/2023

Reviewer name: Tsukasa Fukunaga

Reviewer Comments to Author:

In this study, the authors have developed CheRRI, a machine learning-based software to predict RNA-RNA interactions using large-scale interactome data as the training input. Previous methods for predicting RNA-RNA interactions have relied on the RNA energy models, which had moderate but not high accuracy. The approach of improving prediction accuracy by integrating machine learning with large-scale experimental data and RNA energy models should be an effective strategy, and thus the research direction is highly important. However, this study did not involve sufficient experiments or analysis to demonstrate the usefulness of the software.1.In the fourth paragraph of the Introduction, the authors stated "genome-wide applications are currently out of reach" and "filtering the predictions for functional candidates, specifically on a transcriptome-wide scale, remains an unsolved challenge". I understood that the authors developed CheRRI to solve this problem. CheRRI requires interaction prediction results by IntaRNA as the input, and thus the prediction by IntaRNA is first needed. However, for the large-scale data analysis, the filtering should be performed before the prediction by IntaRNA, which is computationally time-consuming. In other words, this method has not achieved the application of IntaRNA to the transcriptome level. In addition, because there are now methods for predicting RNA-RNA interactions at the transcriptome level, such as RIsearch2 and RIblast, the discussion in the introduction is not sufficiently based on previous research.2. The authors showed that the AUPR of CheRRI is sufficiently higher than the AUPR of MFE in the Fig. 4. However, the authors did not provide the sufficient analysis of which features contributed to the higher accuracy of the predictions. The authors should analyze features that are effective for classification, e.g. through the feature importance analysis and SHAP analysis. In particular, it would be interesting to analyze in what ways humans and mice differ (or have in common). Depending on the features obtained from this analysis, methods such as pre-filtering before the prediction by IntaRNA or incorporating the features to make IntaRNA more accurate should be suggested.3. Because this study used the interaction results from a specific experiment as training data, it may be learning the experimental bias rather than the intrinsic nature of the RNA-RNA interaction. The authors should apply CheRRI to existing RNA-RNA interaction benchmark tests and show that the results obtained with the present method are not experimental bias.

Level of Interest

Please indicate how interesting you found the manuscript: Choose an item.

Quality of Written English

Please indicate the quality of language in the manuscript: Choose an item.

Declaration of Competing Interests

Please complete a declaration of competing interests, considering the following questions:

- Have you in the past five years received reimbursements, fees, funding, or salary from an organisation that may in any way gain or lose financially from the publication of this manuscript, either now or in the future?
- Do you hold any stocks or shares in an organisation that may in any way gain or lose financially from the publication of this manuscript, either now or in the future?
- Do you hold or are you currently applying for any patents relating to the content of the manuscript?
- Have you received reimbursements, fees, funding, or salary from an organization that holds or has applied for patents relating to the content of the manuscript?
- Do you have any other financial competing interests?
- Do you have any non-financial competing interests in relation to this paper?

If you can answer no to all of the above, write 'I declare that I have no competing interests' below. If your reply is yes to any, please give details below.

I declare that I have no competing interests

I agree to the open peer review policy of the journal. I understand that my name will be included on my report to the authors and, if the manuscript is accepted for publication, my named report including any attachments I upload will be posted on the website along with the authors' responses. I agree for my report to be made available under an Open Access Creative Commons CC-BY license (http://creativecommons.org/licenses/by/4.0/). I understand that any comments which I do not wish to be included in my named report can be included as confidential comments to the editors, which will not be published.

Choose an item.

To further support our reviewers, we have joined with Publons, where you can gain additional credit to further highlight your hard work (see: https://publons.com/journal/530/gigascience). On publication of this paper, your review will be automatically added to Publons, you can then choose whether or not to claim your Publons credit. I understand this statement.

Yes Choose an item.
