# Supplementary material for: CheRRI—Accurate classification of the biological relevance of putative RNA–RNA interaction sites
Source: Gigascience. 2024 Jun 5;13:giae022. doi: 10.1093/gigascience/giae022 (PMC11152173; doi:10.1093/gigascience/giae022)
Supplement: giae022_Reviewer_2_Report_Original_Submission — David Mathews -- 12/11/2023 [file giae022_reviewer_2_report_original_submission.pdf]

Reviewer Report

Title: CheRRI - Accurate classification of the biological relevance of putative RNA-RNA interaction sites.

Version: Original Submission **Date:** 12/11/2023

Reviewer name: David Mathews

Reviewer Comments to Author:

In "CheRRI - Accurate classification of the biological relevance of putative RNA-RNA interactions sites," MÃ¼ller et al. report a new machine learning model to classify putative RNA-RNA interactions as true positive or false positive. The method is able to post-process predictions made by thermodynamics to improve the prediction accuracy for RNA-RNA interactions.Minor Revisions:I like the premise of the work. RNA-RNA interaction prediction identifies many false positives, and a machine learning approach to remove false positives is welcome. I have some concerns about presentation that should be revised before publication.The precision-recall curves in Figure 4 compare the free energy of MFE structure prediction to CheRRI for human and mouse. The MFE prediction is not the best baseline; the most accurate methods (including intaRNA) consider the RRI free energy change and the accessibility to binding. Additionally, the negative examples are generated from examples that are "highly scored," which I understood to mean the intaRNA score. In this way, the negative set was designed to be a challenge for free energy methods. To address these concerns, Figure 4 should be generated using the full intaRNA model and not just the MFE free energy as the baseline performance. The text should also clarify that CheRRI is designed to post process the thermodynamic prediction, therefore Figure 4 is not a comparison of two methods, but a demonstration of how CheRRI augments the thermodynamics. The current text suggests that it is a comparison of distinct methods.My second concern is with the loss of performance when transferring the model between organisms (mouse to human and human to mouse). This suggests that the model is not completely transferable. I would have hypothesized that the features would be universal, at least within mammals. There is some cursory discussion that the transferability is not high, but the manuscript should address this as a concern.This work has some similarities to earlier work that identified features for siRNA or miRNA target determination and then used those features to predict target sites [Nat Biotech. 2004. 22: 326; NAR. 2008. 36: 640; NAR. 2013. 41: e138]. This manuscript might cite this prior work and explain that CheRRI is a more general approach for target site prediction.In the supplement, S2 provides a list of features that are used. The list is not completely obvious, and some additional description would be helpful. For example, I assume the Shannon entropy is a mean entropy for nucleotides calculated from base pairing probabilities. However, this is not completely clear, and the text does not state how large a folding region is used for estimating the pair probabilities. Additionally, I assume the terms with GC content use the GC content just for the binding region, but again that is not completely clear.Typo:The quality of writing is excellent. It is clear and concise.I found just one typo: on page 3, "constraint" should be "constrained".

Level of Interest

Please indicate how interesting you found the manuscript: Choose an item.

Quality of Written English

Please indicate the quality of language in the manuscript: Choose an item.

Declaration of Competing Interests

Please complete a declaration of competing interests, considering the following questions:

- Have you in the past five years received reimbursements, fees, funding, or salary from an organisation that may in any way gain or lose financially from the publication of this manuscript, either now or in the future?
- Do you hold any stocks or shares in an organisation that may in any way gain or lose financially from the publication of this manuscript, either now or in the future?
- Do you hold or are you currently applying for any patents relating to the content of the manuscript?
- Have you received reimbursements, fees, funding, or salary from an organization that holds or has applied for patents relating to the content of the manuscript?
- Do you have any other financial competing interests?
- Do you have any non-financial competing interests in relation to this paper?

If you can answer no to all of the above, write 'I declare that I have no competing interests' below. If your reply is yes to any, please give details below.

I declare I have no competing interests.

I agree to the open peer review policy of the journal. I understand that my name will be included on my report to the authors and, if the manuscript is accepted for publication, my named report including any attachments I upload will be posted on the website along with the authors' responses. I agree for my report to be made available under an Open Access Creative Commons CC-BY license (http://creativecommons.org/licenses/by/4.0/). I understand that any comments which I do not wish to be included in my named report can be included as confidential comments to the editors, which will not be published.

Choose an item.

To further support our reviewers, we have joined with Publons, where you can gain additional credit to further highlight your hard work (see: https://publons.com/journal/530/gigascience). On publication of this paper, your review will be automatically added to Publons, you can then choose whether or not to claim your Publons credit. I understand this statement.

Yes Choose an item.
